# Biodiversity of microorganisms in the Baltic Sea: the power of novel methods in the identification of marine microbes

**DOI:** 10.1093/femsre/fuae024

**Published:** 2024-10-04

**Authors:** Hanna Mazur-Marzec, Anders F Andersson, Agata Błaszczyk, Przemysław Dąbek, Ewa Górecka, Michał Grabski, Katarzyna Jankowska, Agata Jurczak-Kurek, Anna K Kaczorowska, Tadeusz Kaczorowski, Bengt Karlson, Marija Kataržytė, Justyna Kobos, Ewa Kotlarska, Beata Krawczyk, Aneta Łuczkiewicz, Kasia Piwosz, Bartosz Rybak, Krzysztof Rychert, Conny Sjöqvist, Waldemar Surosz, Beata Szymczycha, Anna Toruńska-Sitarz, Grzegorz Węgrzyn, Andrzej Witkowski, Alicja Węgrzyn

**Affiliations:** Department of Marine Biology and Biotechnology, University of Gdansk, Al. Piłsudskiego 46, PL-81-378 Gdynia, Poland; Department of Gene Technology, KTH Royal Institute of Technology, Science for Life Laboratory, Tomtebodavägen 23A, SE-171 65 Solna, Stockholm, Sweden; Department of Marine Biology and Biotechnology, University of Gdansk, Al. Piłsudskiego 46, PL-81-378 Gdynia, Poland; Institute of Marine and Environmental Sciences, University of Szczecin, Mickiewicza 16a, PL-70-383 Szczecin, Poland; Institute of Marine and Environmental Sciences, University of Szczecin, Mickiewicza 16a, PL-70-383 Szczecin, Poland; International Centre for Cancer Vaccine Science, University of Gdansk, Kładki 24, 80-822 Gdansk, Poland; Department of Environmental Engineering Technology, Gdansk University of Technology, Narutowicza 11/12, PL-80-233 Gdansk, Poland; Department of Evolutionary Genetics and Biosystematics, University of Gdansk, Wita Stwosza 59, PL-80-308 Gdansk, Poland; Collection of Plasmids and Microorganisms, University of Gdansk, Wita Stwosza 59, PL-80-308 Gdansk, Poland; Laboratory of Extremophiles Biology, Department of Microbiology, University of Gdansk, Wita Stwosza 59, PL-80-308 Gdansk, Poland; Swedish Meteorological and Hydrological Institute , Research and Development, Oceanography, Göteborgseskaderns plats 3, Västra Frölunda SE-426 71, Sweden; Marine Research Institute, Klaipėda University, Universiteto ave. 17, LT-92294 Klaipeda, Lithuania; Department of Marine Biology and Biotechnology, University of Gdansk, Al. Piłsudskiego 46, PL-81-378 Gdynia, Poland; Institute of Oceanology, Polish Academy of Sciences, Powstańców Warszawy 55, PL-81-712 Sopot, Poland; Department of Biotechnology and Microbiology, Gdansk University of Technology, Narutowicza 11/12, PL-80-233 Gdansk, Poland; Department of Environmental Engineering Technology, Gdansk University of Technology, Narutowicza 11/12, PL-80-233 Gdansk, Poland; National Marine Fisheries Research Institute, Kołłątaja 1, PL-81-332 Gdynia, Poland; Department of Environmental Toxicology, Faculty of Health Sciences with Institute of Maritime and Tropical Medicine, Medical University of Gdansk, Dębowa 23A, PL-80-204 Gdansk, Poland; Pomeranian University in Słupsk, Arciszewskiego 22a, PL-76-200 Słupsk, Poland; Environmental and Marine Biology, Åbo Akademi University, Henriksgatan 2, FI-20500 Åbo, Finland; Department of Marine Biology and Biotechnology, University of Gdansk, Al. Piłsudskiego 46, PL-81-378 Gdynia, Poland; Institute of Oceanology, Polish Academy of Sciences, Powstańców Warszawy 55, PL-81-712 Sopot, Poland; Department of Marine Biology and Biotechnology, University of Gdansk, Al. Piłsudskiego 46, PL-81-378 Gdynia, Poland; Department of Molecular Biology, University of Gdansk, Wita Stwosza 59, PL-80-308 Gdansk, Poland; Institute of Marine and Environmental Sciences, University of Szczecin, Mickiewicza 16a, PL-70-383 Szczecin, Poland; University Center for Applied and Interdisciplinary Research, University of Gdansk, Kładki 24, 80-822 Gdansk, Poland

**Keywords:** Baltic Sea, marine ecosystem, diversity of microorganisms, molecular methods, prokaryotic and eukaryotic microorganisms, marine viruses

## Abstract

Until recently, the data on the diversity of the entire microbial community from the Baltic Sea were relatively rare and very scarce. However, modern molecular methods have provided new insights into this field with interesting results. They can be summarized as follows. (i) Although low salinity causes a reduction in the biodiversity of multicellular species relative to the populations of the North–East Atlantic, no such reduction occurs in bacterial diversity. (ii) Among cyanobacteria, the picocyanobacterial group dominates when considering gene abundance, while filamentous cyanobacteria dominate in means of biomass. (iii) The diversity of diatoms and dinoflagellates is significantly larger than described a few decades ago; however, molecular studies on these groups are still scarce. (iv) Knowledge gaps in other protistan communities are evident. (v) Salinity is the main limiting parameter of pelagic fungal community composition, while the benthic fungal diversity is shaped by water depth, salinity, and sediment C and N availability. (vi) Bacteriophages are the predominant group of viruses, while among viruses infecting eukaryotic hosts, *Phycodnaviridae* are the most abundant; the Baltic Sea virome is contaminated with viruses originating from urban and/or industrial habitats. These features make the Baltic Sea microbiome specific and unique among other marine environments.

## Teaser

The specific Baltic Sea hydrographic conditions and significant anthropogenic pressure make the composition, diversity, and abundance of microorganisms unique relative to other marine habitats.

## Introduction

### Baltic Sea characterization

**Figure 1.**
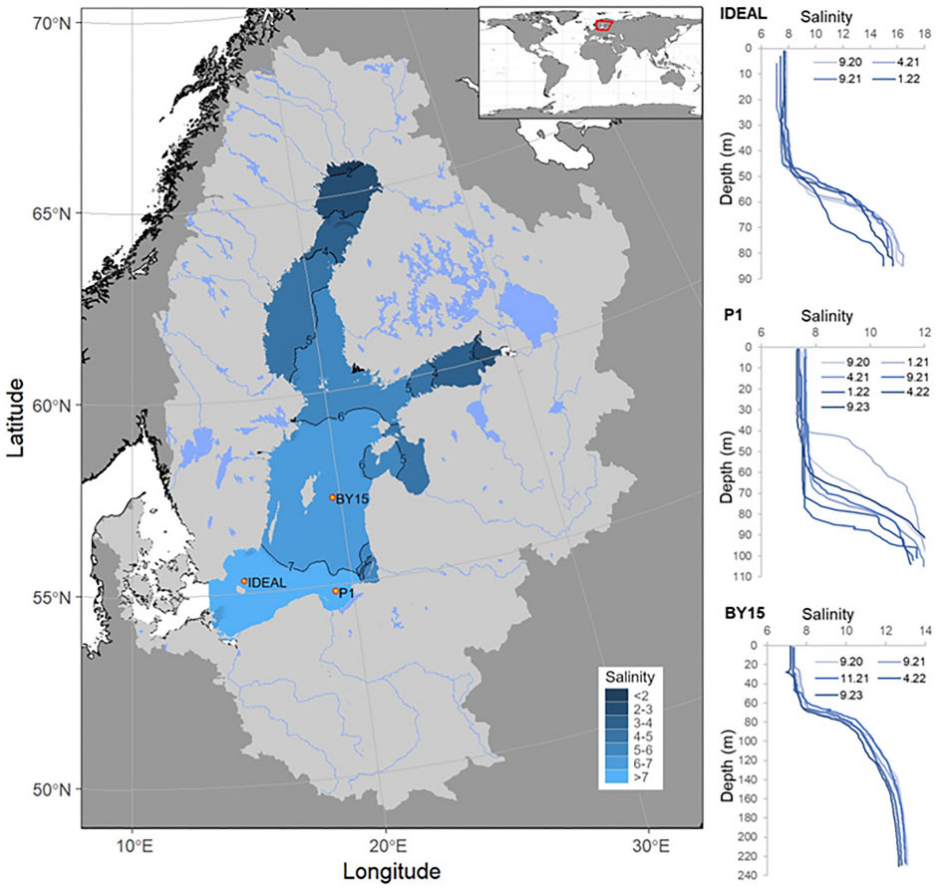
Map of the Baltic Sea with the surface salinity and catchment area, and indication of major deeps. Salinity levels are indicated, with a scale representing regions of the sea with corresponding concentrations of NaCl. The catchment is shown as an area around the sea region. Vertical distributions of salinity at Bornholm (IDEAL), Gdansk (P1), and Gotland (BY15) Deeps within 2020–2023 (Szymczycha et al. 2024) are indicated.

The Baltic Sea is a semienclosed shelf sea located in northern Europe. The sea is connected to the North Sea by the narrow and shallow waters of the Sound and the Belt Sea, the Kattegat, and the Skagerrak, forming a transition area to the North Sea. Its catchment area, inhabited by about 85 million people, is four times larger than the sea surface (Fig. 1). The biogeochemical functioning of the brackish Baltic Sea is directly linked to its location, specific topography, and hydrographic settings, and it is influenced by eutrophication, deoxygenation, acidification, and anthropogenic pollution (Kuliński et al. 2022). Therefore, the composition, diversity, and abundance of the microorganism community are affected by the surface salinity gradient from marine to nearly freshwater, reduced circulation, and strong stratification causing hypoxic and anoxic conditions in bottom waters, high sedimentation rates with high organic matter content, and inflow of terrestrial microorganisms (Xu et al. 2022).

### Research on Baltic microorganisms

Initially, research on Baltic microbes focused on phytoplankton. Such research has been carried out for more than 100 years (Cleve 1889, Hensen 1887, 1890, Aurivillius 1896, Apstein 1905, Fraude 1907, Lakowitz 1907, Lohmann 1908, 1911, Schulz 1926). Hensen and collaborators started modern plankton research by developing sampling nets and quantitative calculation methods. They used them during expeditions in 1883–1886 in the western parts of the Baltic and North Seas (Finni et al. 2001). In 1902, the International Council for the Exploration of the Seas (ICES) established international coordination of plankton research, with sampling four times a year throughout the entire Baltic Sea area. Lohmann (1908, 1911) used a hand centrifuge to concentrate plankton and showed the importance of the nanoplankton (planktonic microbes of 2–20 µm of cell diameter/size). He concluded that net sampling leads to overlooking and underestimating small plankton. Quantitative analyses of nanoplankton and microplankton (planktonic cells of 20–200 µm diameter/size) started when the sedimentation chamber method was established (Utermöhl 1931, 1958). Papers by Apstein (1905), Driver (1908), Merkle (1910), and Krabbi (1913) contributed to the Helsinki Commission (HELCOM) program in 1974. They were the basis for intensifying cooperation among countries surrounding the Baltic Sea (Finni et al. 2001). Pliński and coworkers had a significant impact on the present phytoplankton research by constructing the identification keys of phytoplanktonic species from the southern Baltic Sea that helped in their correct taxonomic classification (Pliński and Komarek 2007, Pliński and Hindak 2010, Pliński and Witkowski 2020). Jochem (1988, 1989) showed the importance of phototrophic picocyanobacteria of the *Synechococcus* genus.

Nevertheless, until recently, comprehensive studies on Baltic microorganisms were relatively scarce. The number of published works on the Baltic Sea microorganisms increased significantly only since the 1990s, with the development of molecular and bioimaging methods. Until the 1980s, microbiological research in the Baltic Sea was carried out using classical techniques with microbial cultures. The main research center was the Institute of Oceanography at the University of Kiel (Germany), where bacteria involved in the nitrogen cycle were studied (Rheinheimer 1967), and microorganisms new to science were described (Ahrens and Moll 1970). Research has also been conducted on the link between algal blooms and bacterial populations (Rieper 1976). The problem of eutrophication and its relationship with the presence of microorganisms (Hoppe 1978) and the direct connection between 33 parameters describing physical, chemical, and biological characteristics were processed with particular reference to microbiological activity (Bölter et al. 1981). Methodological works and comparisons of the presence of bacteria in different areas of the Baltic Sea were carried out in Finland (Tvärminne Zoological Station) (Väätänen 1977, 1980). The isolation and identification of bacteria in polluted areas were addressed in the Swedish Water and Air Pollution Research Institute in Stockholm, (Neilson 1980). In 1983, the first comprehensive study of the composition of bacterioplankton from the Baltic Sea and Skagerrak was conducted employing epifluorescence and electron microscopy (Schmaljohann 1984). In 1985, a study was published showing that in freshwater (Lake Hodges, California) and brackish water (Baltic Sea), the release and uptake of orthophosphate were tightly coupled (Ammerman and Azam 1985). At that time, the vertical distributions of microorganisms were also analysed in different areas of the Baltic Sea using culture and microscopic methods (Rheinheimer et al. 1989). Subsequent years brought about research on the production and respiration of overall plankton and ultraplankton communities (Kuprinen 1987, Lignell 1990). Stable isotope labeling of nitrogen sources was used to investigate nitrogen flows in the sea (Sörensson et al. 1989). Subsequent use of secondary ion mass spectrometry allowed to determine nitrogen uptake into individual cells (Klawonn et al. 2016, Olofsson et al. 2019).

Thanks to evolving genetic methods, four strains of budding hyphal bacteria, which had very similar chemotaxonomic properties, were isolated from the Baltic Sea (Schlesner et al. 1990, Brettar and Rheinheimer 1991). Dominant marine bacterioplankton species were identified among colony-forming bacteria (Pinhassi et al. 1997). At the end of the 20th century, HELCOM activities led to the reduction of dangerous pollutants which, in turn, caused the regeneration of flora and fauna in some areas (Rheinheimer et al. 1989). However, new threats have begun to be recognized. In this respect, the turning point occurred with the introduction of molecular biology techniques to study microorganisms in the Baltic Sea ecosystem. Therefore, this review article focuses on modern genetic and genomic methods that have revolutionized our understanding of the biodiversity of Baltic microbes.

## Importance of modern methods, including genetic and genomic analyses and advanced imaging techniques, in studies on marine microorganisms

### Metagenomics in analyses of microorganisms—advantages and limitations

With the advances of metagenomics (Handelsman et al. 1998), exploring the richness and diversity of microorganisms, including those thriving in marine environments, became more straightforward than ever. The approach to studying genetic material recovered directly from environmental samples without the need to isolate and culture individual microbes revolutionized our understanding of microbial communities in terms of taxonomy and phylogeny. It shed new light on the role of microorganisms in the environment (Gilbert and Dupont 2011). Metagenomics, with its ever-growing and evolving methodology, has also challenged traditional ideas about microbial evolution, showing that horizontal gene transfer and other processes can shape microbial genomes not only in the case of closely related organisms but that such transmission can also occur between distant taxonomical groups (Miller et al. 2005). The picture that emerges from metagenomic analyses allows us to see a multiplex network of interactions between different microorganisms and between microorganisms and their environment that can contribute to a better understanding of the whole system and such complex processes as nutrient cycling and bioremediation (Shu and Huang 2022).

One of the key advantages of metagenomics is the possibility of discovering new species and functional genes that were previously unknown or difficult to study (Kodzius and Gojobori 2015). This notion has led to the development of new concepts and hypotheses about microbial ecology and evolution that have challenged many old assumptions and paradigms (Gilbert and Dupont 2011, Alves et al. 2018). The discovery of proteorhodopsin-based photoheterotrophy (Beja et al. 2000) and ammonia-oxidizing archaea (AOA) (Nicol and Schleper 2006) are outstanding examples of metagenomics potential to examine microbial function. Metagenomics has also revealed that microbial communities are much more diverse and complex than previously thought. In this regard, two landmark studies have to be mentioned. They were conducted within the Sargasso Sea Project (Venter et al. 2004) and the Global Ocean Sampling (GOS) expedition (Rusch et al. 2007). The first effort produced a dataset comprising over 10^9^ DNA sequence reads assembled into 1800 unique genomes. The study resulted in the identification of 48 unknown bacterial phylotypes and 1.2 million previously unknown genes. However, a recent survey on global functional analysis of 26 931 metagenomes revealed an even more complex landscape with 1.17 billion protein-coding sequences with no similarity to any sequences deposited in the protein families database (Pfam) (Pavlopoulos et al. 2023).

The second project, GOS, focused on the biogeography and diversity of specific bacterial groups. It provided information on the potential for marine microbes to produce newly identified biological compounds with commercial and medical applications. Other initiatives that followed, including the spectacular Tara Oceans Project, focused on understanding the ocean ecosystem, particularly in light of climate change (Sunagawa et al. 2020), and continued these large-scale pioneering studies. One of the recent initiatives was devoted to harnessing metagenomics to bioprospect viruses from extreme habitats (Aevarsson et al. 2021). A high prevalence of genes without assigned functions was observed. In the case of archaeal viruses, ~75% of genes in analysed genomes showed no homology to genes in other viruses or cellular organisms (Liu et al. 2019). The same was noted for thermophilic bacteriophages isolated from hydrothermal vents in Iceland (Aevarsson et al. 2021), which makes viruses from extreme habitats a valuable source of unknown genes.

Despite its simplicity and extraordinary potential, metagenomics suffers from some limitations, starting with sample preparation and sequencing and ending with data analysis (Gilbert et al. 2010, Dopheide et al. 2019, van der Loos and Nijland 2020, Mioduchowska et al. 2022, Olenyi et al. 2023). The severe challenge concerns high-throughput next-generation sequencing (NGS), which results in an enormous amount of raw data. Indeed, NGS facilities provide more data than users can analyse, interpret, and share (Chistoserdova 2010, Wong 2018). This challenge requires state-of-the-art infrastructure for metagenomic research and novel data curation and analysis approaches. In Europe, the exemplary initiative is ELIXIR (https://elixir-europe.org/), started in 2013 with a focus on a single, coordinated infrastructure devoted to curating life science data, training, analysis, and facilitating collaboration between member institutes and researchers (Harrow et al. 2021). This initiative is significant in the case of marine metagenomics as the ELIXIR provides databases such as MarRef (prokaryotic genomes), MarDB (incomplete sequenced prokaryotic genomes), MarCat (genes from marine metagenomic samples), and METdb (microeukaryotic marine species transcriptomes) that are reference data sources for interpretation and species assignment purposes (Robertsen et al. 2018, Klemetsen et al. 2018, Niang et al. 2020). Recently, the KAUST Metagenomic Analysis Platform (KMAP) Global Ocean Gene Catalog 1.0 was established, with 317.5 million gene clusters, being the most extensive open-source catalog to study the diversity and function of microbes (Laiolo et al. 2024). The following subsections present some primary modern molecular methods used in studies on microorganisms, including those from the Baltic Sea.

### Analysing metagenome-assembled genomes

An established dogma since the mid-1980s specifies that only about 1% of all microbes may be cultivated under laboratory conditions (Staley et al. 1982, Staley and Konopka 1985). Even though this conception was challenged later on (van Teeseling and Jogler 2021), we continue to fall short in cultivation and thus only investigate microbial diversity and function on a limited scale. As argued previously (Salcher and Šimek 2016, Vilanova and Porcar 2016), cultivation-based techniques are required for a holistic understanding of microbial species, their biology, and ecology. However, investigations using metagenome-assembled genomes (MAGs) may provide opportunities for novel insights into the diversity and function of uncultivated taxa and reduce the knowledge gap between model and nonmodel organisms.

MAGs are reconstructed de novo through “binning,” i.e. grouping assembled contigs that share high sequence composition similarity and display similar coverage profiles across samples (Albertsen et al. 2013, Alneberg et al. 2014). This results in assembling genomes of varying completeness and purity. For example, in their venture to expand the tree of life, Parks et al. (2017) reconstructed 7903 bacterial and archaeal genomes using publicly available metagenome data. Half of these were ≥90% complete with ≤5% contamination. However, recent development of methods focused on advancing the quality of reconstructed MAGs has considerably improved completeness and resolved contamination issues (Vollmers et al. 2022).

A few studies utilizing MAGs from the Baltic Sea have been published in recent years. The first one provided many bacterioplankton genomes from the central Baltic Sea, several representing species new to science with no previous sequences in existing databases (Hugerth et al. 2015). A global comparative analysis of these genomes revealed significant fragment recruitment from brackish waters in North America but only minor from lake or ocean areas, suggesting the existence of a global brackish metacommunity. Comparative analyses of MAGs from the Caspian and Baltic Sea (Mehrshad et al. 2016) also revealed high similarity between brackish populations. In a subsequent study, MAGs were reconstructed from many samples spanning significant environmental gradients of the Baltic Sea, resulting in genomes of 350 different prokaryotic species (Alneberg et al. 2020). That report demonstrated that these species’ distributions along various niche gradients (e.g. salinity and depth) could be roughly predicted based solely on the functional genes present in their genomes. A phylogenomic analysis, including thousands of MAGs originating from freshwater, brackish (from the Baltic and Caspian Seas), and marine environments, confirmed the existence of a brackish-specific metacommunity and revealed that most brackish lineages transitioned from freshwater or marine lineages millions of years ago, i.e. long before the Baltic Sea was formed (Jurdzinski et al. 2023).

A population genomic framework can be applied, allowing for intraspecific comparative analysis of genomes for further, in-depth investigation of MAGs. Such an approach may reveal how microbial populations are genomically structured across space and time and imply what factors in the organisms’ environment regulate their diversity. The intraspecies diversity of uncultivated Baltic Sea MAGs has been further investigated and correlated significantly with environmental factors like salinity and temperature (Sjöqvist et al. 2021). Moreover, specific genes were discovered within several MAGs that adapted to different salinity regimes, enabling the species to exist across a wide range of salinities (Sjöqvist et al. 2021). Using more traditional sequence-based approaches, like the comparison of ribosomal RNA genes (for example, 16S rRNA), would not enable the detection of such intraspecies variability. Functional genes in benthic MAGs from the Baltic Sea environment have also been shown to differ across environmental gradients, and they can be further modified by resource availability (Broman et al. 2022). This suggests that variability in various parameters, e.g. oxygen, salinity, and nutrient content, can influence metabolic pathways in benthic MAGs.

### DNA barcoding

DNA barcoding is a powerful technique for efficient and cost-effective characterization and monitoring of prokaryotic and eukaryotic communities in ecosystems. It is, therefore, widely used in biodiversity studies of marine environments. It relies on using short and standardized genome regions, known as “DNA barcodes,” as the reference taxonomy molecular markers for comparison with the sequence of corresponding DNA regions of unknown specimens. While the concept is based upon the fact that genetic differences between species tend to be greater than those within a species, the choice of DNA sequence used for barcoding is critical for reliable taxonomy identification as distinct regions of DNA may have different mutation rates and patterns of variation.

Metabarcoding, taking advantage of high-throughput sequencing (HTS) of a specific DNA marker, enables taxonomic identification of multiple species present in a sample. It involves four steps: (i) genetic material extraction from environmental samples, (ii) PCR (polymerase chain reaction)-based amplification of a specific variable region of DNA flanked with conserved regions that can be used for primers annealing; (iii) high-throughput DNA sequencing, and (iv) data analysis using a reference database.

The barcoding procedure is straightforward and requires standard laboratory equipment except for the NGS platform. The most critical part concerns the amplification of the target variable regions. In the case of eukaryotes, including microbial eukaryotes, the target gene is the one coding for 18S rRNA that possesses hypervariable regions like V1–V2 (Mohrbeck et al. 2015), V4 (Lejzerowicz et al. 2015), V7 (Guardiola et al. 2015), V8 (Günther et al. 2018), and V6–V8 or V9 (Amaral-Zettler et al. 2009, Latz et al. 2022). However, 18S rRNA does not resolve all eukaryotic plankton to the species level, as the variability between species can be too low. Thus, the internal transcribed spacer (ITS) and the 28S regions are also utilized (Latz et al. 2022). The term “long read sequencing” is often used when 18S, ITS, and 28S are sequenced. For zooplankton, as for other animals, the standardized target sequence is a fragment (ca. 650 bp) of mitochondrial gene *COI* coding for cytochrome *c* oxidase subunit I (Folmer et al. 1994, Herbert et al. 2002). For eukaryotic microalgae, as for other plants, the standardized two-locus barcode is used; one is a fragment (ca. 600 bp) of the plastid gene coding for ribulose 1,5-bisphosphate carboxylase (*rbcL*), while another is a fragment (ca. 1550 bp) of the maturase (*matK*) gene (Chase et al. 2007). The barcode for fungi is the nuclear ITS of the ribosomal DNA (Seifert 2009). Prokaryotes, such as bacteria and archaea, are detected using the 16S rRNA gene; however, its use is limited for some genera due to a low potential in differentiating between closely related microbes (Zeigler 2003, Janda and Abbott 2007). Functional groups of prokaryotes can be targeted with primers specific to their marker genes, for instance, dinitrogenase reductase gene *nihF* for diazotrophs (Gaby et al. 2018) or photosynthetic reaction center small subunit *pufM* gene for anoxygenic phototrophs (Gazulla et al. 2023).

Unlike cellular organisms, no universal marker gene targets are available for viruses. However, this inconvenience can be overcome by using genes targeting common groups of viruses. This approach was applied in studies on the seasonality of arctic viral communities (Filee et al. 2008, Larsen et al. 2008). T4-like bacteriophages were captured with gene *g23*, while the *mcp* gene was used for large dsDNA phytoplankton viruses. Both genes mentioned above code for viral major capsid proteins.

In conclusion, for more complicated analysis, the standard barcode set may not be sufficient to distinguish between closely related species or identify species from certain groups. In such cases, it is necessary to use additional DNA regions as barcodes or develop new barcoding markers (Antil et al. 2023). One of the valuable databases is the BOLD system, which stores all the published primers used for barcoding purposes (http://boldsystem.org/index.php/public_Primer_PrimerSearch). The PCR-amplified DNA targets are sequenced using HTS technologies generating millions of short or long reads, depending on the platform used (Illumina/Nanopore). This step is followed by quality control and filtering to remove low-quality reads, adapter sequences, and contaminants, such as host DNA or sequencing artifacts. The remaining reads are then analysed using bioinformatic tools, such as (i) Barcode of Life Data Systems (http://www.boldsystems.org/), which offers various applications for data management, quality control, and analysis, as well as a reference library of barcode sequences for many taxonomic groups (Ratnasingham and Herbert 2007); (ii) QIIME2, which is a bioinformatic software package for microbiome analysis that also includes tools for DNA sequence analysis, taxonomic assignment, and diversity analysis (Bolyen et al. 2019), (iii) mothur (https://mothur.org/), which offers a variety of methods for analysing microbial communities, including operational taxonomic units (OTU)-based clustering, phylogenetic analysis, examining amplicon sequence variants (ASVs), and network analysis (Schloss et al. 2009). Many researchers now use ASVs rather than OTUs, employing the DADA2 algorithm (Callahan et al. 2016). The choice of software depends on the research question, the taxonomic group of interest (bacteria, archaea, viruses, or eukaryotes), and the user’s level of expertise.

### Digital PCR

Digital PCR (dPCR) is a method for quantifying nucleic acids that involves dividing a sample into a large number of minor reactions in the form of droplets (Vogelstein and Kinzler 1999). With this method, the absolute quantity of the target can be determined without the need for a calibration curve or reference sample (Hindson et al. 2011). The droplet generator, part of the dPCR platform, uses microfluidic channels to merge the aqueous and oil phases in a controlled manner, creating stable droplets of uniform size (less than one nanoliter volume of each) and composition. Each droplet contains either one copy, a few copies, or no molecules of the target sequences. Depending on the instrument used, it is possible to process ca. 20 000–1 000 000 separate PCR reactions simultaneously in a single microtube (Hindson et al. 2011, Dobnik et al. 2016). The PCR reaction then proceeds within each droplet, with the fluorescent dyes allowing for real-time detection and quantification of the amplification products. The nucleic acid quantity is measured in the final step by placing the tube in a dPCR droplet reader, which uses fluorescence to distinguish between positive and negative droplets. By counting the number of positive and negative droplets, the absolute concentration of the target DNA can be calculated using Poisson statistics (Hindson et al. 2011, Pinheiro et al. 2012).

The information obtained from the dPCR analysis can be used to calculate the concentration of the target DNA in the original sample. Because dPCR relies on partitioning the sample into many individual reactions, it can provide greater accuracy and precision than traditional real-time quantitative PCR (qPCR), which typically measures the average signal from bulk samples. dPCR makes it possible to detect and quantify rare or low-abundance targets that may be difficult to detect using regular qPCR. Additionally, dPCR is more tolerant to inhibitors and can detect and quantify low-abundance targets with higher sensitivity than traditional PCR.

### Bioimaging methods

Bioimaging methods that visualize the structure and function of living cells and their communities have long been used to study morphology, organization, and functions of microalgae and bacteria. For example, the accumulation of fluorescent carcinogenic or mutagenic organic compounds in microalgae can be followed by confocal fluorescent microscopy (Subashchandrabose et al. 2014), and the development of the various stages of the consortium can be observed with a scanning electron microscope (Mu et al. 2021).

These methods, combined with the robustness of flow cytometry (FC), provide a superb instrument for a variety of applications in marine studies. FC has been implemented to identify and quantify different phytoplankton species at the level of species or even strain (Dunker 2019, Fuchs et al. 2022) and track their abundance and community structure changes. This information can be applied to monitor ecosystem health, predict harmful algal blooms, and study the effects of environmental stressors on phytoplankton populations.

### Imaging FlowCytobot

Imaging FlowCytobot (IFCB) is a submersible instrument used to monitor and analyse *in situ* phytoplankton in aquatic environments (Sosik and Olson 2007). IFCB combines automatic sampling, FC, and digital imaging techniques to capture high-resolution images of individual phytoplankton cells and particles and analyse their size, shape, and fluorescence characteristics. A built-in high-speed flow cytometer passes a water sample through a flow cell, where a laser beam (635 nm wavelength) illuminates each cell or particle. The light scattered and emitted by the cells and particles is then collected and analysed by multiple digital cameras and sensors, which capture detailed images of each object.

Chlorophyll fluorescence measurement allows for the discrimination between heterotrophic and phototrophic cells in taxa. IFCB can analyse large numbers of particles and cells in real-time, providing rapid and high-throughput analysis of phytoplankton populations (Sonnet et al. 2022). IFCB can detect cells up to 150 µm in size (Sonnet et al. 2022) and identify a wide range of phytoplankton species, including diatoms and harmful algae, like filamentous cyanobacteria in the Baltic Sea (Kraft et al. 2021). The measurements provide valuable information for water quality and ecosystem health assessment. The instrument proved to be a powerful platform for studying the dynamics and diversity of phytoplankton communities with utmost precision and accuracy (Daskova et al. 2017).

## Exploring Baltic Sea microbial communities: overview of the sampling campaigns

Marine microbial communities consist of all three domains of life, namely bacteria, archaea, and single-celled eukaryota. Microorganisms isolated using standard cultivation methods are rarely numerically dominant in the communities from which they were obtained. Instead, they are isolated due to their ability to rapidly grow into colonies on high-nutrient artificial growth media, typically under aerobic conditions at moderate temperatures (Hugenholtz 2002). Moreover, the vast majority of marine microorganisms remain unexplored, primarily due to limitations in current cultivation techniques (Amann et al. 1995, Hugenholtz 2002). The taxonomic and functional diversity of the global ocean microbiome has been revealed by employing technological advances in sampling, DNA sequencing, and bioinformatics, especially with Tara Ocean expeditions (Sunagawa et al. 2020, Tara Ocean Foundation et al. 2022) and other expeditions (the bioGEOTRACES project, the Micro B3 project, the Bio-GO-SHIP project, the Marine Biofilms, and others) (Lu et al. 2023).

The Integrated Ocean Drilling Program (IODP) expedition collected 347 cored sediments from different settings of the Baltic Sea, covering the last glacial–interglacial cycle (Andrén et al. 2015). Recent multidisciplinary approaches combining multiple omics technologies (metagenomics, metatranscriptomics, metaproteomics, and metabolomics) applied to environmental samples with cell biology, imaging, ecosystem modeling, remote sensing, and oceanography have significantly improved our understanding of individual microbes and their roles in the global ocean (DeLong and Karl 2005). Furthermore, in the last few years, many thousands of MAGs have been reported for various environments and host-associated microbiota (Setubal 2021). The OceanDNA MAG catalogue lists over 50 000 prokaryotic genomes originating from different marine niches (Nishimura and Yoshizawa 2022), including the Baltic Sea microbes (Hugerth et al. 2015, Alneberg et al. 2018a), with several single amplified genomes (Alneberg et al. 2018b).

Understanding the key processes that control bacterial community composition has enabled predictions of bacterial distribution and function within ecosystems (Herlemann et al. 2016). The Baltic Sea offers an exceptional ecosystem for studying the influence of environmental factors and gradients on microbiome community diversity and functions (Lindh and Pinhassi 2018). Baltic Sea microbes are unique, highly diverse, and adapted to brackish-water ecosystems (Ininbergs et al. 2015). Since most microbiomes are sensitive to environmental disturbances (Lindh and Pinhassi 2018), this review presents a selection of research concerning biodiversity changes in response to environmental gradients and anthropogenic pressure. It discusses the role of Baltic microorganisms in biogeochemical cycles and host–microbiome interactions, underscoring their importance to the field.

## Bacteria (excluding cyanobacteria) and archaea in the Baltic Sea

### Biodiversity in response to environmental gradients

The overall taxonomic diversity and activity of bacteria and archaea are ruled by different gradients (e.g. nutrient availability, pH, and temperature). However, it is salinity that has a significant impact on the composition of the microbial community (Oren 2002, Lozupone and Knight 2007, Auguet et al. 2010, Chen et al. 2021). In the Baltic Sea, the intermediate salinity conditions cause a reduction of the general biodiversity. Multicellular species are especially less abundant and less differentiated in their genetic material, relative to the populations of the North–East Atlantic (Johannesson and André 2006). In contrast, no reduction in bacterial diversity at brackish conditions can be observed (Herlemann et al. 2011, Dupont et al. 2014, Hugerth et al. 2015, Herlemann et al. 2016). This could be concluded based on the first detailed description of an autochthonous brackish microbiome (Herlemann et al. 2011), which was corroborated by further studies (discussed below).

Ultra-deep Illumina sequencing (with an average of 10^5^ sequences/sample) of rRNA gene amplicons of surface water, sampled in summer along a 2-km transect following the salinity gradient (Hu et al. 2016), showed similar features to those observed in previous studies (Herlemann et al. 2011, 2016, Dupont et al. 2014). Specifically, the bacterial community composition changed gradually along the salinity gradient, and the difference in community composition (beta-diversity) was significantly correlated with the difference in salinity (Hu et al. 2016). Similar patterns were observed at the Vistula River mouth (Gołębiewski et al. 2017). Moreover, studies on cable bacteria (*Desulfobulbaceae* that couple sulfide oxidation to oxygen reduction over centimeter distances by mediating electric currents) confirmed that salinity significantly influenced species richness and composition (Dam et al. 2021). Cable bacteria can limit sulfide release by promoting iron oxide formation in sediments. For example, such an activity of cable bacteria was observed in spring in the Gulf of Finland, possibly explaining why bottom waters in this highly eutrophic region rarely contain sulfide in summer (Hermans et al. 2019). Berner et al. (2018) studied the response of summer plankton communities to increased temperature and reduced salinity in the Gotland Deep, belonging to the Baltic Proper (the part of the Baltic Sea located between the Åland Sea and the Danish Straits). They found that shifts in community composition of heterotrophic bacteria are influenced directly by abiotic factors (temperature and salinity) and potentially indirectly by cyanobacteria (Berner et al. 2018). Based on pyrosequencing of 16S rRNA genes from surface waters taken from eight stations extending from the Gulf of Finland to the southern Baltic Sea, von Scheibner et al. (2018) concluded that bacterial activities are suppressed during early phytoplankton blooms at low temperatures and are not substantially altered by short-term warming events.

A long-term model system with natural fluctuations (providing support for future climate change-related shifts) revealed that with increasing temperatures, enhanced growth, and earlier appearance of picocyanobacteria during the summer occurred. As a consequence, the diversity of microbial communities in bottom waters decreases (Seidel et al. 2022). Such conditions can cause higher oxygen consumption on the sediment surface, resulting in an increase of anaerobic processes closer to the sediment surface and higher diversity in the sediments.

Bacterioplankton inhabiting the Baltic Proper does not consist of locally adapted freshwater or marine populations but rather contains members of a global brackish metacommunity that most likely adapted to brackish conditions before the Baltic Sea was formed (Riemann et al. 2008, Hugerth et al. 2015). Large-scale phylogenomic analysis of freshwater, brackish, and marine quality-filtered MAGs revealed that bacterial species rarely exist in multiple biomes. In contrast, distinct brackish basins cohosted numerous species, but their intraspecific population structures displayed clear signs of geographic separation (Jurdzinski et al. 2023). One of the core bacterial populations newly identified in the metagenomic datasets obtained from Baltic Sea surface waters was SAR11 subclade IIIa (SAR11-IIIa) (Vidanage et al. 2020). SAR11, an Alphaproteobacterial clade (Pelagibacterales), is a group of small, carbon-oxidizing, pelagic bacteria that are among the most abundant in aquatic environments, accounting for about 25% of the plankton cells in the ocean photic zone (Morris et al. 2002, Giovannoni 2017). SAR11-IIIa was the major driver facilitating the seasonal shifts in the overall community structure over the brackish waters of the Baltic Sea (Vidanage et al. 2020). Using CARD-FISH methodology, Herlemann et al. (2014) revealed that SAR11-IIIa was abundant in oligohaline–mesohaline conditions (salinities 2.7–13.3), with maximal abundances at a salinity of 7 (up to 35% of total bacteria, quantified with a universal bacterial probe EUB). As expected, SAR11-I/II was abundant (27% of EUB) in the marine parts of the Baltic Sea, whereas freshwater lineage SAR11-IIIb was below the detection limit at all stations. The shift from SAR11-IIIa to SAR11-I/II was also confirmed in the vertical salinity gradient in the deeper basins of the Baltic Sea (Herlemann et al. 2014). On the other hand, freshwater lineage SAR11-IIIb and other freshwater bacteria were observed in the Gulf of Gdansk (Piwosz et al. 2013).

The complex changes in adaptation to osmotic shock (changes in salinity) and cold stress (temperature changes) were also shown in transcriptomic and proteomic analyses of *Shewanella baltica*, a significant player in denitrification and bioremediation in the marine environment (Kloska et al. 2020, 2022). Adaptation to salinity changes involved the accumulation of DNA-binding proteins and increased polyamine uptake, probably to coat and protect the nucleoid to counteract adverse changes in DNA topology due to ionic shifts (Kloska et al. 2022). Exposing *S. baltica* to low temperatures revealed changes in the bacterial transcriptome (studied with RNA sequencing, validated with the RT–qPCR method), especially massive downregulation of gene expression, which covered about 70% of differentially expressed genes (Kloska et al. 2020).

As indicated above, the Baltic Sea is one of the largest anthropogenically induced hypoxic areas in the world. High nutrient input to the system and low water exchange result in eutrophication and oxygen depletion below the halocline (Thureborn et al. 2016, Broman et al. 2017). The influence of oxygen and depth gradient on biodiversity changes has often been reported (Labrenz et al. 2007, Brettar et al. 2012, Thureborn et al. 2016, Nishimura and Yoshizawa 2022). In Landsort Deep, strong stratification was observed across a depth transect, encompassing both functional capacity and taxonomic affiliation. Functional capacity demonstrated the closest correlation with key environmental parameters such as oxygen level, salinity, and temperature (Thureborn et al. 2013). Oxygen-depleted Landsort Deep sediments were active, as indicated in metatranscriptomic studies, and metabolic pathways for carbon transformation, including fermentation, dissimilatory sulfate reduction, and methanogenesis, were found to be predominant (Thureborn et al. 2016).

Bacteria from the Epsilonproteobacteria group were shown to be mainly responsible for chemoautotrophic activity in the dark CO_2_-fixation maxima of the Black Sea and central Baltic Sea redoxclines (Grote et al. 2008). Pyrosequencing of 16S rRNA genes from sediment samples obtained from Skagerrak and Bothnian Bay revealed that the community structure matches the redox stratification with the known Fe-reducers, coinciding with the zone of Fe-reduction. *Desulfobulbaceae, Desulfuromonadaceae, Pelobacteraceae, Desulfobacter*, and *Geobacter*, known to reduce Fe ions, were detected and showed the highest abundance near the Fe(III)/Fe(II) redox boundary (Reyes et al. 2016). S-, Fe-, and Mn-reducers were also detected by others (Edlund et al. 2008, Sinkko et al. 2011, Vandieken et al. 2012). In 2014, the exceptional, major Baltic inflow brought saline and oxygenated water into the anoxic, sulfidic deep basins of the central Baltic Sea and caused dramatic changes in the composition of bacterial species. After this event, the uplifting of the formerly anoxic bacterial community and the dominance of Epsilonproteobacteria was recorded (Bergen et al. 2018).

Community structures of active bacterial populations are mainly influenced by organic carbon content in coastal Baltic Sea sediments. Other important factors included total nitrogen and redox potentials, as studied along a vertical redox profile (three redox depths: 179, −64, and −337 mV) using terminal-restriction fragment length polymorphism (T-RFLP) and 16S rRNA gene sequencing of clone libraries (Edlund et al. 2008). The results of those studies were obtained with two techniques studying metabolically active and growing bacteria: (i) analysis of reverse-transcribed 16S rRNA from total RNA extracts, and (ii) immunocapture of BrdU-labeled DNA from DNA extracts from the same sediment samples (Edlund et al. 2008). Bacterial community structures in Baltic Sea sediments appeared similar along small horizontal spatial scales if the environmental variables were relatively constant, but these similarities decreased with increasing geographical distances (Edlund and Jansson 2006, Edlund et al. 2006, 2008). In the Gulf of Bothnia, sediments’ distinct depth stratification of archaeal and bacterial taxa was noted (Rasigraf et al. 2020).

Salinity is also a significant factor in structuring the bacterio-benthos, and there is no loss of bacterial richness at intermediate salinities. The bacterial communities of marine, mesohaline, and oligohaline sediments differed in terms of the relative rRNA abundances of the major bacterial phyla/classes (Klier et al. 2018). Other studies confirmed the previous assumptions that the active community differed mainly from the total community, based on the analyses of amplicon sequencing of RNA and DNA fractions in sediments (Edlund et al. 2008) and in water (Miettinen et al. 2019).

The mean catabolic per-cell rate of microorganisms drops gradually with depth transitioning to life in slow motion, typical for the deep biosphere (Jørgensen et al. 2020). Under energy-limited conditions, the continuous synthesis and breakdown of proteins are metabolically expensive for microorganisms. Thus, the deep biosphere microbial community conserves energy by repairing existing biomolecules. This was confirmed by metagenomic and metatranscriptomic analyses of sediments. The samples were retrieved during the IODP Expedition 347 (Andrén et al. 2015) from Landsort Deep and the Little Belt in the Baltic Sea (covering the period from the Baltic Ice Lake ca. 13 000 years ago to the present). These analyses indicated the high abundance, taxonomically widespread occurrence, and expression of genes encoding the protein-l-isoaspartate (d-aspartate) *O*-methyltransferase enzyme (PCMT). PCMT recognizes damaged l-isoaspartyl and d-aspartyl residues in proteins and catalyses their repair. This protein-maintenance trait was more prevalent in the Baltic deep subsurface than in pure laboratory cultures (Mhatre et al. 2019). Moreover, Bird et al. (2019) investigated sediments with single-cell genomics, metabolomics, metatranscriptomics, and enzyme assays to identify possible subsistence mechanisms employed by uncultured Atribacteria, Aminicenantes, Actinomycetota group OPB41, Aerophobetes, Chloroflexi, Deltaproteobacteria, Desulfatiglans, Bathyarchaeota, and Euryarchaeota marine group II lineages (Bird et al. 2019). Atribacteria and Chloroflexi were also identified as active community members (with targeted sequencing of 16S rRNA transcripts) (Zinke et al. 2017).

The subsurface life under extreme energy limitation is facilitated by the exploitation of recalcitrant substrates, by biochemical protection of nucleic acids and proteins, and by repair mechanisms for random mismatches in DNA or damaged amino acids in proteins (Jørgensen et al. 2020). Some functions appeared to be shared by multiple lineages, such as trehalose production and NAD-consuming deacetylation, both of which have been shown to increase cellular life spans by stabilizing proteins and nucleic acids, respectively (Bird et al. 2019). Relative abundance of genes conferring salinity tolerance was found to correlate with the present Baltic salinity, even in deep late-glacial sediment layers where salinity has increased since the sediment was deposited in a “freshwater lake” phase of the Baltic Sea (the Ancylus Lake) over 9000 years ago (Marshall et al. 2018). Metatranscriptomic analysis identified methane cycling, sulfur cycling, and halogenated compound utilization as active *in situ* respiratory metabolisms. Genes for cell maintenance, division, motility, and antimicrobial production were also transcribed. This indicated that microbes in deep subsurface Baltic Sea basin sediments were alive and thriving (Zinke et al. 2017).

Many studies on bacterial community composition in the Baltic Sea did not distinguish between particle-associated (PA) and free-living (FL) bacteria or neglected the PA fraction by prefiltration, removing most particles. Rieck et al. (2015) studied surface samples of three Baltic Sea stations (marine, mesohaline, and oligohaline) in two different seasons (summer and fall/winter). Amplicon sequencing of the 16S rRNA gene revealed significant differences in the community structure of both bacterial fractions among stations and seasons, with an exceptionally high fraction of bacterial OTUs found exclusively in the PA samples. Therefore, to gain a deeper understanding of the diversity and dynamics of aquatic bacteria, both PA and FL fractions of bacteria should be studied independently (Rieck et al. 2015). When studying the dynamics of microbial food webs over a yearly cycle in the Baltic Sea Proper, Martínez-García et al. (2022) revealed seasonal changes in both the composition of the bacterial community in the PA and FL size fractions and their contribution to organic matter utilization and carbon cycling. Furthermore, higher relative quantities of *hgcAB* genes were found in metagenomes from marine particles compared to FL communities in anoxic water, suggesting that such particles constitute hotspot habitats for Hg methylators in oxygen-depleted seawater (Capo et al. 2020).

### Biodiversity in biogeochemical cycles

Bacteria and archaea mediate most transformations in the cycling of nitrogen, phosphorus, trace metals, and other nutrients (Falkowski et al. 2008). Numerous studies on biodiversity in the context of biogeochemical cycles were conducted. Due to the unique characteristics of the Baltic Sea, the majority of research has been devoted to the nitrogen and carbon cycle with particular emphasis on methane transformations.

Biological N_2_ fixation is the oceans’ main source of fixed nitrogen (N) (Gruber 2005). The Baltic Sea receives large nitrogen inputs from diazotrophic (N_2_-fixing) heterocystous cyanobacteria, but the significance of heterotrophic N_2_ fixation was also documented (Farnelid et al. 2013). The diversity, abundance, and transcription of the *nifH* gene, encoding the nitrogenase enzyme, in suboxic and anoxic waters of the Baltic Sea (Baltic Proper, Gotland Deep, and Bornholm Basin) were studied with 454 pyrosequencing and qPCR. Since heterotrophic diazotrophs are not limited by light or water temperature, they could potentially fix N_2_ in the pelagial. Several *nifH* phylotypes were abundant in suboxic/anoxic waters during all sampling years in the above mentioned basins (Farnelid et al. 2013).

Reconstruction of the nitrogen cycle at Landsort Deep indicated a potential for syntrophy between archaeal ammonium oxidizers and bacterial denitrification at anoxic depths. In contrast, anaerobic ammonium oxidation genes were absent despite substantial ammonium levels below the chemocline (Thureborn et al. 2013). In sediments of the Bothnian Sea, the dominant roles of denitrification and only minor anammox or DNRA were confirmed. Interestingly, a higher diversity of anammox bacteria was detected using metagenomic data than with a PCR‐based technique (Rasigraf et al. 2017). The high genomic potential for complete denitrification to N_2_ was noted in those sediments but the importance of anaerobic ammonium oxidation and dissimilatory nitrite reduction to ammonium was minor. Genomic potential for aerobic ammonia oxidation was dominated by Thaumarchaeota (Rasigraf et al. 2017). Metatranscriptomes retrieved from pelagic suboxic zones of the central Baltic Sea (Gotland Deep and Landsort Deep) with an *in situ* fixation system using an automatic flow injection sampler resulted in the detection of thaumarchaeal ammonia monooxygenase transcripts that were up to 30-fold more abundant than those detected in samples obtained using standard oceanographic sampling systems (Feike et al. 2012). In the Bothnian Sea sediments, the potential for nitrogen cycling was dominated by reductive processes via a truncated denitrification pathway, encoded exclusively by bacterial lineages (Rasigraf et al. 2020).

AOA are an important component of the planktonic community, linking nitrogen and carbon cycles through nitrification and carbon fixation (Francis et al. 2005). By enriching AOA from a brackish, oxygen-depleted water-column in the Landsort Deep, Berg et al. (2015) performed seawater batch experiments. Those were based on phylogenetic analyses of the 16S rRNA and the ammonia monooxygenase subunit A (*amoA*) gene sequences, revealing an affiliation with assemblages from low-salinity and freshwater habitats, with “*Candidatus* Nitrosoarchaeum limnia” as the closest relative (Berg et al. 2015).

Methane in the seabed is mainly oxidized to CO_2_ with sulfate as the oxidant before it reaches the overlying water column, and microbial oxidation occurs within the sulfate–methane transition (SMT) zone (Beulig et al. 2019). Sedimentary gene pools suggested that nearly all potential methanogens within and beneath the SMT zone belonged to uncultured archaea of the Methanomicrobia group, namely anaerobic methane clade ANME-1, typically associated with anaerobic methane oxidation (Laso-Pérez et al. 2023). Analysis of a MAG suggested that predominant ANME-1 has the enzymatic potential to catalyse both methane production and consumption (Beulig et al. 2019). In the sediments of the Bothnian Sea, the abundance of methanotrophic archaea of the ANME-2a clade was related to the presence of methane and varied with sediment iron content (Rasigraf et al. 2020). Pockmarks are important “pumps” proposed to play a significant role in global methane cycling and harboring a unique assemblage of diverse prokaryotes (Iasakov et al. 2022). Recently, a large pockmark with active gas seepage and submarine groundwater discharge was discovered in the central Gulf of Gdansk, southern Baltic Sea. Within this pockmark, the composition of prokaryotes atypical for marine surface sediments resulted from the combination of freshwater and high organic matter content, and reflected active *in situ* methanogenesis (Idczak et al. 2020).

Massive sequencing of the 16S rRNA gene V4 hypervariable regions for the samples from thirteen pockmark horizons (the Baltic Sea) collected at depths from 0 to 280 cm below seafloor revealed that members of the phyla Planctomycetota, Chloroflexota, Desulfobacterota, Caldatribacteriota, Acidobacteriota, and Pseudomonadota predominated across all horizons, comprising 58.5% of the total prokaryotic community (Iasakov et al. 2022). Shifting of the ANME Archaea subclusters depending on depth (from 2a to 2b subcluster, which was predominant in sulfate-rich upper horizons, including SMT zone and together with sulfate-reducing bacteria) had a primary role in aerobic oxidation of methane, coupled with sulfate reduction. Furthermore, this led to shifting to ANME-1a and ANME-1b, which alone mediated the anaerobic oxidation of methane or switching to methanogenic metabolism (Iasakov et al. 2022). This reflects a tendency for niche separation.

### Biodiversity upon the influence of anthropogenic pressure

Marine ecosystems, especially coastal areas, are subject to progressively increased anthropogenic disturbances (Halpern et al. 2008). Numerous studies have proven the impact of point sources of pollution on microbial biodiversity (Nogales et al. 2011). In the Baltic Sea, the impact of point sources of anthropogenic pressure (e.g. river mouths, aquacultures, harbors, ports, and wastewater treatment plant outfalls) on microbial life is especially pronounced due to the specific hydrogeographic properties (Fig. 1), where any external influence is powerful because of the semienclosed character of this sea.

Sinkko et al. (2011) revealed that bacterial community composition differed horizontally from the river estuary receiving agricultural phosphorus to the open sea, and vertically from the surface to deeper sediment layers, mainly along the gradient of nutrients (tested with the T-RFLP and cloning and sequencing of the 16S rRNA gene obtained from sediments from Paimionjoki river estuary to the open sea—the Baltic Proper, Western Gulf of Finland). The study conducted on fish farms in the Turku Archipelago (northern Baltic Sea) showed that medium-scale fish farming in shallow water seemed to have a greater impact on sediment bacteria than fish farming in open water, and has the potential to enrich the presence of fish and human pathogens (Tamminen et al. 2011). Metabarcoding also provided evidence for the influence of aquaculture-associated eutrophication on sediment communities (Harrison et al. 2021). Differences in bacterial biodiversity (studied with 16S rRNA amplicon sequencing) were also noted in biofilms on different types of microplastic (Kesy et al. 2019), and benthic community structure influenced by recreational boating (Iburg et al. 2021).

In Baltic seawater microcosm experiments, diesel fuel, crude oil, and shale oil influenced the hydrocarbon-degrading bacterial community (Viggor et al. 2013). Research-based on 16S rRNA amplicon sequencing of the surface water and sediment samples in oil-contaminated sites showed that the composition of archaeal communities was ecosystem- and season-dependent. Oil contamination significantly altered the composition and network properties of archaeal communities in the littoral sediment (Yan et al. 2018). A set of phenol-degrading bacterial strains isolated from Baltic Sea surface water were mainly affiliated with the *Pseudomonas* and *Acinetobacter* genera. However, that study also widened the range of phenol-degraders by including the genus *Limnobacter*. Furthermore, using an NGS approach, the genes coding for the largest mPH subunit of phenol hydroxylase of *Limnobacter* strains were found to be the most prevalent ones in the microbial community of the Baltic Sea surface water (Vedler et al. 2013). Using the Baltic Sea Reference Metagenome (BARM) dataset, Capo et al. (2020) studied the abundance and distribution of the genes encoding proteins involved in Hg methylation (the *hgcAB* gene cluster). The *hgcAB* genes were predominantly detected in anoxic water, but some *hgcAB* genes were also detected in hypoxic and normoxic waters. Phylogenetic analysis identified putative Hg methylators within Deltaproteobacteria in oxygen-deficient water layers, as well as Spirochaetota-like and Kiritimatiellaeota-like bacteria. Higher relative quantities of *hgcAB* genes were found in metagenomes from marine particles, compared to FL communities in anoxic water, suggesting that such particles are hotspot habitats for Hg methylators in oxygen-depleted seawater. Studies on the gut microbiota of copepods (with qPCR for *hgcA*) led to the conclusion that endogenous Hg methylation occurs in zooplankton and may contribute to seasonal, spatial, and vertical MeHg variability in the water column and food webs (Gorokhova et al. 2020).

The microbiota of cod *Gadus morhua callarias* L., inhabiting the chemical munition dump site (Bornholm Deep), was significantly less taxonomically diverse compared to those from a nonpolluted reference site (Wilczyński et al. 2022). On the contrary, using culture-dependent molecular techniques, Hantula et al. (1996) revealed that the bacterial community was more divergent in a polluted location than in clean areas (Hantula et al. 1996). Moreover a comparison of microbiomes from the long-term oil-polluted coastal site and less exposed sites revealed that those sites had a similar potential for petroleum hydrocarbon degradation (Miettinen et al. 2019). However, transcriptome analysis of the Baltic Sea model bacterium *Rheinheimera* sp. BAL341, upon the influence of organic pollutants (batch experiments with environmentally relevant concentrations), revealed significant shifts in gene expression profiles compared with controls in exponential growth (Karlsson et al. 2019). Analysis of metagenomes and metatranscriptomes to detect metacaspases, involved in stress response and programmed cell death in bacteria and phytoplankton, accounted for ∼4% of the bacteria (mainly Bacteroidota, Alpha- and Betaproteobacteria, and Cyanobacteria). The gene abundance was significantly higher in larger or PA bacteria (>0.8 µm), and filamentous Cyanobacteria dominated metacaspase gene expression throughout the bloom season (Asplund-Samuelsson et al. 2016).

Altered microbial communities in human-impacted marine environments can, in turn, have detrimental effects on human health (i.e. the spread of pathogens and antibiotic resistance) (Nogales et al. 2011). Since water environments are reservoirs of antibiotic resistance (Zhang et al. 2009, Zheng et al. 2021b), one can consider the ocean as a global reservoir of both clinically relevant and potentially newly discovered antibiotic resistance genes (ARGs) (Hatosy and Martiny 2015, Xu et al. 2023). Studies in this field were also conducted in the Baltic Sea, mainly using cultivation techniques coupled with standard molecular methods (Mudryk 2005, Mudryk et al. 2010, Moskot et al. 2012, Tiirik et al. 2014, Kotlarska et al. 2015, Łuczkiewicz et al. 2015, Gross et al. 2022a,b). Other important reservoirs for antibiotic resistance are wastewater treatment plants (Łuczkiewicz et al. 2010, Laht et al. 2014, Kotlarska et al. 2015, Pazda et al. 2020), river mouths (Sadowy and Łuczkiewicz 2014, Kotlarska et al. 2015), and farming or wildlife, especially birds (Literak et al. 2010, Dreyer et al. 2022, Rybak et al. 2022, Gross et al. 2022b). The fish farm resistomes were enriched in transposon and integron-associated genes and ARGs encoding resistance to antibiotics used to treat fish at the farms (Muziasari et al. 2016). In contrast, the total relative abundance values of ARGs were higher in the control sediment resistome, and there were mainly genes encoding efflux pumps, followed by beta-lactam resistance genes, which are found intrinsically in many bacteria. This suggests that there is a natural Baltic sediment resistome (Muziasari et al. 2016). Interestingly, Muziasari et al. (2017) analysed the composition of the antibiotic resistome from the intestinal contents of 20 fish from the Baltic Sea farms, using a high-throughput method, WaferGen qPCR array with 364 primer sets, to detect and quantify ARGs, mobile genetic elements, and the 16S rRNA gene. That study suggested that feces from farmed fish contribute to resistome enrichment in farm sediments despite the lack of contemporaneous antibiotic treatments at the farms. The intestinal contents of individual farmed fish had their own resistome compositions (Muziasari et al. 2017). Moreover, it was proven (using PCR) that the increase in the prevalence of tetracycline resistance genes is caused by the persistence of these genes in the absence of selection pressure (Tamminen et al. 2011). The ARGs persisted in sediments below fish farms (northern Baltic Sea, Finland) at very low antibiotic concentrations during the 6-year observation period from 2006 to 2012. Although the ARGs persisted in the farm sediments, they were less prevalent in the surrounding sediments (Muziasari et al. 2014). Since studying the mobility of ARGs in environmental resistomes is challenging, Pärnänen et al. (2016) developed a low-cost and labor-efficient method based on inverse PCR and long-read sequencing for studying the mobility potential of environmental resistance genes. Analysis of metagenomes and metatranscriptomes of size-fractionated bacterial communities for the presence of mobile elements showed transposase levels up to 1.7% of bacterial genes, and 2% of bacterial transcripts. This suggests an elevated rate of transposition-based genome change and host adaptation (Vigil-Stenman et al. 2017).

Another important aspect concerns the occurrence of possible human pathogens in the coastal region of the Baltic Sea. Several studies have been carried out on the presence of bacteria belonging to *Escherichia coli* and other fecal coliforms, *Aeromonas, Enterococcus, Pseudomonas, Staphylococcus*, and *Vibrio* genera (Eiler et al. 2006, Sadowy and Łuczkiewicz 2014, Bier et al. 2015, Kotlarska et al. 2015, Łuczkiewicz et al. 2015, Gyraite et al. 2019, Fleischmann et al. 2022, Gross et al. 2022a, Kalvaitienė et al. 2024). Since microplastics in aquatic environments provide novel habitats for surface-colonizing microorganisms, it was found that *Vibrio* spp. are early colonizers of microplastic surfaces in the Baltic Sea mesocosm (Kesy et al. 2019). Therefore, it is clear that contaminations derived from urban and industrial sources greatly impact the microbial diversity in the Baltic Sea, including (but not restricted to) bacterial species that are harmful to humans and resistant to many antibiotics. These issues of biological contamination with pathogens and a rising problem with antibiotic resistance is also discussed later in this paper, in a chapter devoted to contaminations in the Baltic Sea.

### Microbiomes associated with Baltic Sea organisms

The marine microbiome refers to all marine microorganisms, including bacterioplankton, microorganisms associated with organic and inorganic particles, and those living on and inside marine animals, plants, and macroalgae (Mioduchowska et al. 2022). Studies on microbes associated with other Baltic Sea organisms are mostly based on cultivation, coupled with molecular analyses, mainly 16S rRNA gene sequencing (e.g. bacteria associated with the sponge *Halichondria panicea*, Althoff et al. 1998; the brown algae *Laminaria saccharina*, Wiese et al. 2009; bryozoans, Heindl et al. 2020; *Fucus vesiculosus* and *Delesseria sanguinea*, Goecke et al. 2013; soft coral *Alcyonium digitatum*, Pham et al. 2016; *Nodularia spumigena*, Toruńska-Sitarz et al. 2018; farmed mussels *Mytilus* spp., Utermann et al. 2018; ascidian *Ciona intestinalis*, Utermann et al. 2020, 2021; European plaice *Pleuronectes platessa*, Ghotbi et al. 2022; herring *Clupea harengus*, Huotari et al. 2022). Many studies have also been devoted to the occurrence of pathogens in the Baltic Sea, with particular attention given to marine mammals (Siebert et al. 2001, Sonne et al. 2020) or birds (Dreyer et al. 2022, Schick et al. 2022, Gross et al. 2022a). However, with the development of HTS, it became possible to analyse the microbiota of other Baltic organisms.

The gut microbiota of *G. morhua callarias* L. from the chemical munition dump site was disturbed, compared to those from a nonpolluted reference site. Moreover, taxa associated with fish diseases (e.g. *Vibrionaceae* and *Aeromonadaceae*) were more prevalent, and probiotic taxa (Actinomycetota, *Rhodobacteraceae*) were less frequent in the guts of individuals from the dump site (Wilczynski et al. 2022). The gut microbiota of wild Baltic salmon parr (a subpopulation of Atlantic salmon *Salmo salar* L.) differs from those of wild North- and East-Atlantic salmon parr, probably due to biogeographical differences or host-selective pressures (Skrodenytė-Arbačiauskienė et al. 2022). Comparative microbiome analysis of indigenous and nonindigenous gelatinous zooplankton species in the low-saline southwestern Baltic Sea, conducted by Jaspers et al. (2020), also showed significant differences in microbiome compositions. The overall differentiation between microbiomes was driven by eight indicator OTUs, which included *Mycoplasma* and *Vibrio* species. These bacteria can be problematic, as they include known pathogens relevant to human health and aquaculture activities (Jaspers et al. 2020).

The coexistence of bacteria and protozoa in aquatic environments has led to the evolution of predatory defense mechanisms in the former group. Some of the predation-resistant bacteria are also pathogenic to humans and other mammals. During field studies (using amplicon sequencing of 16S rRNA of predation-resistant bacteria and 18S rRNA of bacterivorous protozoa), conducted in the coastal area of the northern Baltic Sea, co-occurrence patterns were found (such as *Legionella* and *Ciliophora*). Moreover, the interactions were genotype-specific, as indicated, for example, in *Rickettsia*. The predation-resistant bacteria sequence diversity was larger in bays and freshwater inlets compared to offshore sites, indicating local adaptations (Eriksson et al. 2022). The 16S amplicon sequencing of the gut microbiota of the sand lances (*Ammodytes tobianus* and *Hyperoplus lanceolatus*) from the North Sea and the Baltic Sea revealed that the relative abundance of Gammaproteobacteria may be driven by their relative abundance in the surrounding environment. However, in the case of the Alphaproteobacteria and Actinomycetota, relative abundances might depend more strongly on host species (Fietz et al. 2018).

In samples collected in the Baltic Sea and the adjusted area (the Kattegat, Skagerrak, and the eastern North Sea), the *Ulva*-associated bacterial composition (resolved with Oxford Nanopore sequencing of full-length 16S rRNA gene) was strongly structured by both salinity and host species. The largest shift in the bacterial consortia coincided with the horohalinicum (defined as the transition zone from freshwater to marine conditions), with salinity values of 5–8 practical salinity unit (PSU). Characteristic bacterial communities associated with distinct salinity regions may, therefore, facilitate host adaptation across the environmental gradient (van der Loos et al. 2022). The diversity of the tunic-associated microbiota differed also from that of the ambient seawater samples and between sampling sites (Utermann et al. 2020). Wäge et al. (2019) have successfully used the microcapillary technique to sample the gut microbiome of two copepods (*Temora* sp. and *Acartia* sp.), which commonly dominate the surface and subsurface waters of the central Baltic Sea. The 16S rRNA gene amplicon sequencing revealed differences among the dominant bacterial communities associated with *Temora* sp. (Actinomycetota, Betaproteobacteria, and Flavobacteria) and *Acartia* sp. (Actinomycetota, Alphaproteobacteria, and Betaproteobacteria), and the surrounding water (Proteobacteriota, Cyanobacteria, and Verrucomicrobia), but also intraspecific variability was recorded. This research also revealed indicative species for methane production pathways (Wäge et al. 2019). Quantifying bacterial and archaeal 16S rRNA and the *mcrA* gene within copepod faecal pellets using droplet dPCR showed a small number of methanogenic Archaea, while *mcrA* transcripts indicating methanogenic activity were not detected. This suggests that copepod faecal pellets from the central Baltic Sea, similar to analogous data on copepod guts (Wäge et al. 2019), have potential but are unlikely hotspots for methane production by methanogenic archaea (Wäge et al. 2020).

Gorokhova et al. (2020) studied the occurrence of the bacterial gene *hgcA*, in the guts of dominant zooplankters in the Northern Baltic Sea (copepods *Acartia bifilosa, Eurytemora affinis, Pseudocalanus acuspes* and *Limnocalanus macrurus*, and cladocerans *Bosmina coregoni maritima* and *Cercopagis pengoi*). All copepods were found to carry *hgcA* genes belonging only to Deltaproteobacteria and Bacillota. Therefore, studies combining population genomics with gut microbial data have the potential to gain insight into an organism’s ecological adaptive capabilities (Fietz et al. 2018).

Another aspect of microbiome research concerns the production of biologically active compounds, such as antimicrobials (Wiese et al. 2009, Heindl et al. 2020, Goecke et al. 2013). This kind of research, linked to metabolomics, has made it possible to reveal a large variety of bioactive compounds compared to conventional screening methods. Ghotbi et al. (2022) used a culture-dependent survey, followed by molecular identification of microbiota associated with the gills and the gastrointestinal tract of European plaice (*P. platessa*). The untargeted metabolomic approach showed the high chemical diversity of cultures of selected bacterial and fungal isolates (Ghotbi et al. 2022). Using UPLC-MS/MS-based metabolomics, a high chemical diversity was also detected among bacterial species associated with *C. intestinalis*. Peptides and polyketides were the predominant classes, but many unknown compounds were also found (Utermann et al. 2020, 2021). Fiorini et al. (2022) proposed to combine high-throughput cultivation techniques with metabolomics to capture and assess the enormous metabolic potential of previously uncultured bacteria (Fiorini et al. 2022). Lee et al. (2015) proposed a pipeline for the functional characterization of multiple metagenome samples (FCMM) to infer major functions as well as their quantitative scores in a comparative metagenomics manner (Lee et al. 2015). Another promising approach was used by Massing et al. (2023), which involved manifold learning, converting the taxonomic time series (obtained through 16S rRNA amplicon sequencing of seawater samples from western Baltic Proper) into information about bacterial metabolic niches. The obtained results prove that manifold learning can broaden our understanding of the links between community composition and its function (Massing et al. 2023).

**Figure 2.**
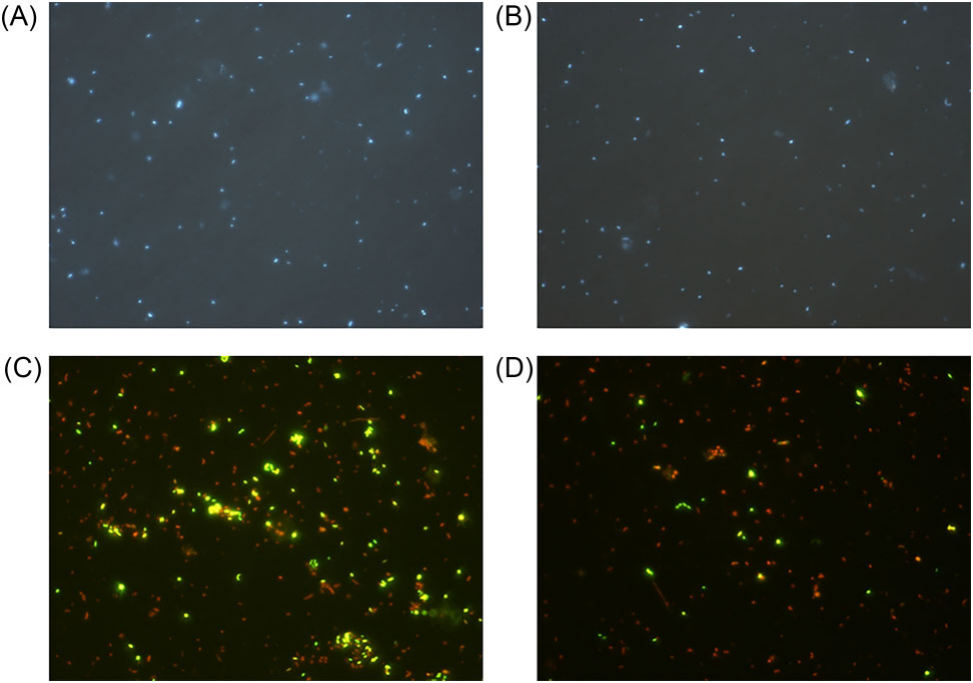
Epifluorescent microphotographs of bacterial cells from surface coastal waters of the Baltic Sea. DAPI (4',6-diamidino-2-phenylindole dye) staining is shown in panels A and B. The live/dead staining (with SYTO 9 and propidium iodide dyes), where alive cells and dead cells are marked by fluorescence, is shown in panels C and D, respectively. Photographs taken by Ewa Kotlarska.

In summary, the diversity of Baltic bacteria living with other organisms is relatively broad, showing that specific interactions between bacteria and animals, plants, and other organisms are important phenomena to be considered when assessing the composition and function of the Baltic microbiome. Examples of microphotographs of Baltic bacteria are shown in Fig. 2.

## Cyanobacteria in the Baltic Sea

The origin of life is generally assumed to be 3.4 billion years ago, but before cyanobacteria started to produce oxygen about 2 billion years ago, all organisms were strictly anaerobic. In fact, cyanobacteria are oxygenic phototrophs (OPs) that play a fundamental role in marine productivity and energy flow. Furthermore, the secondary metabolites produced by these microorganisms affect the diversity of other marine inhabitants. As some taxa produce toxic compounds and form mass blooms, cyanobacterial diversity is also a matter of concern for agencies responsible for water quality and management. Due to potential harmful effects, the first studies on Baltic cyanobacteria (since the 19th century) were primarily focused on filamentous, bloom-forming genera [*Nodularia, Aphanizomenon*, and *Dolichospermum* (former *Anabaena*)]. In the 21st century, studies with the application of advanced techniques have revealed that those taxa might not be the only important players in the Baltic cyanobacterial community.

### Replacing the classical approach (microscopy) with novel methods—the case of monitoring studies

The blooms of filamentous cyanobacteria were recorded in the Baltic as early as in 1854 (Lindström 1855). The exploration of the diversity of this group of Baltic microorganisms started at the end of the 19th century, when Victor Hensen launched phytoplankton studies at Kiel University (Finni et al. 2001). On a larger scale, these studies were intensified in the 1960s (Finni et al. 2001). With the establishment of the HELCOM Phytoplankton Expert Group (PEG, referred to as EG PHYTO from 2022 onwards) in 1991, a preliminary list of Baltic cyanobacterial species began to be published regularly. Thirteen years later, the first checklist of Baltic phytoplankton, including cyanobacteria, was published (Hällfors 2004). Since then, the checklist has been updated annually and is available at https://helcom.fi/helcom-at-work/projects/peg/- “biovolume” file, supported by an image gallery at www.nordicmicroalgae.org. The current catalog contains 110 taxa identified at the species level (accessed on 31st March 2024). The regular monitoring studies are performed by trained taxonomists who use a standardized light microscopy method (CEN 2015), making the data comparable locally and internationally.

While microscopy-based identification and quantification of the cyanobacterial species are essential in Baltic ecosystem bioassessment programs, the use of only these optical methods has many disadvantages: (i) detection of picocyanobacteria is limited, (ii) cryptic and rare species are excluded, (iii) environmental conditions may bias cyanobacterial morphology, (iv) the method is time-consuming, and (v) shortage of specialists using microscopy-based methods has been observed during recent years. Environmental DNA metabarcoding can address some limitations of microscopy in cyanobacterial identification and can increase diversity estimates. For the first time, this method was implemented in Baltic phytoplankton monitoring by Finnish and Swedish researchers (Jerney et al. 2022, Karlson et al. 2022). The results of the pilot studies, both involving sequence analyses of the V4 variable region in the 16S rRNA gene, showed that picocyanobacterial, overlooked in standard monitoring, dominate the Baltic cyanobacterial community when considering relative gene abundance (though filamentous cyanobacteria dominate when assessing the biomass as a criterium). This issue is detailed in the subsequent section.

### Shedding light on the smallest—the importance of picocyanobacteria

Picocyanobacteria (PCy) are a polyphyletic group of small (between 0.2 and 2.0 µm diameter) microorganisms. Metagenomic studies revealed that two genera, *Prochlorococcus* and *Synechococcus*, dominate the phytoplankton in oligotrophic parts of the oceans, whereas *Synechococcus* and *Cyanobium* representants are predominant in brackish and freshwaters (Farrant et al. 2016, Jasser and Callieri 2017). Picocyanobacterial populations are often divided according to their pigment content into phycocyanin (PC)-rich, and phycoerythrin (PE)-rich types. Phylogenomic and ecogenomic studies group marine *Synechococcus*/*Cyanobium* into well-defined cluster 5, which is further divided into three subclusters (SC): SC5.1, composed of marine and a few brackish strains, the closest relatives to oceanic *Prochlorococcus* spp.; SC5.2, the most diverse subcluster in terms of geographic origin and habitat of the strains; and SC5.3, containing both open ocean and freshwater strains (Larsson et al. 2014, Cabello-Yeves et al. 2022, Callieri et al. 2022). Regardless of the method used and the parameter measured, data from the last 20 years indicate that PCy can significantly contribute to the Baltic phytoplankton biomass during the summer. Based on the direct counts by fluorescence microscopy, it was estimated that PCy constitutes 20%–97% of the Baltic cyanobacterial biomass (Mazur-Marzec et al. 2013). When carbon or chlorophyll *a* content was used as a prox,y PCy were determined to make up to 83% of phytoplankton biomass (Zufia et al. 2021 and references therein).

Metabarcoding studies also revealed that picocyanobacterial sequences can dominate in constructed libraries. Different variable regions of the 16S rRNA gene were analysed in those studies: V1–V2 (Xu et al. 2022), V3–V4 (Bertos-Fortis et al. 2016, Hu et al. 2016, Celepli et al. 2017), and V5–V7 (Zufia et al. 2022). The conclusions were as follows: (i) temperature has the dominant influence on the abundance of PCy (positive correlation), (ii) the horizontally varying contribution of SC51.-5.3 individual genotypes strongly depend on salinity, with the most significant shift between 13 and 16 PSUs, and (iii) the Baltic PCy populations are of freshwater origin. The presence of rare taxa, such as microorganisms closely related to the oceanic *Prochlorococcus* populations (SC5.1), was noted only when metagenome sequencing was used; in the case of 16S rRNA amplicon sequencing the taxa were not detected (Celepli et al. 2017).

Analyses of the metagenome-assembled *Synechococcus* genome classified it to the CS5.2 subcluster, revealing the presence of a novel gene cluster encoding proteins for a unique set of light-harvesting antennae (Larsson et al. 2014). This indicated that there may be an as-yet uncharted gene pool in the Baltic Sea. The unique genotype mentioned above dominates the PCy community in the Baltic Proper (on average 88% of cyanobacterial reads; Hu et al. 2016). The Baltic PCy community is composed of both PC-rich and PE-rich taxa, with the dominance of the former in coastal waters. As reported by Zufia et al. (2022), in the future, PC-rich populations may gain a higher proportion of the PCy biomass, and their presence will have a significant impact on the carbon cycle and energy flow. PC-rich cells inhibit the activity of filter-feeders and possess protective mechanisms against predation and viral infections. The impact of PCy on the Baltic ecosystem and biochemical cycles may be even stronger in the future. The abundance of these microorganisms is predicted to increase due to climate change (Flombaum et al. 2013).

### Diversity shift under the wave of climate change and methodological evolution

The diversity of Baltic cyanobacteria is often analysed in the context of gradients of various abiotic parameters, in water masses (horizontally and vertically), and in time. In some cases, it is difficult to assess to what extent the implementation of new techniques has influenced our understanding of cyanobacterial diversity in the Baltic Sea. This is because of the ongoing ecosystem changes related to climate fluctuations (Meier et al. 2022). Some of the problems mentioned above can be solved using modern approaches to explore the trends.

In paleontological studies, analyses of different proxies preserved in the sediments enabled the researchers to look beyond the observation periods. Most investigations conducted in the Baltic Sea area are based on the analyses of fossil pigments. Although the pigments did not allow us to infer the taxonomic composition of cyanobacterial communities, they revealed that cyanobacteria have been present in the Baltic at least since the beginning of the brackish period, which started some 8000 years ago. Nonetheless, the analysis of specific lipids (Sollai et al. 2017, Kaiser et al. 2020), cyanopeptides (Cegłowska et al. 2018), and genetic markers (PC-IGS; Cegłowska et al. 2018) made it possible to trace the history of occurrence of specific genera, species or even subpopulations. The conclusion linking all these studies concerns the dominant effect of temperature on the mass growth of diazotrophic cyanobacteria. However, more research into the past occurrence is needed to understand the present cyanobacterial diversity and to predict future changes.

Global data unequivocally demonstrate that climate change associated with higher solar radiation, rising surface water temperatures, and the subsequent vertical stratification leads to the intensification of cyanobacteria blooms and their expansion into new locations (Zepernick et al. 2022). An abiotic parameter that already strongly differentiates cyanobacterial communities horizontally and which is also strongly dependent on climate change is salinity. In a few projects, cyanobacterial diversity was analysed using nested PCR, amplicon sequencing (16S rRNA), or metagenome sequencing of water samples collected from all over the Baltic area. In those studies, salinity was found to be the factor that most strongly determined the diversity of cyanobacteria (Dupont et al. 2014, Celepli et al. 2017, Reeder et al. 2022). By screening the V1–V2 variable region of 16S rRNA sequences retrieved from Baltic water profiles (3–155 m), Xu et al. (2022) concluded that cyanobacterial reads dominate the upper 8 m of the sea. In the same work, qPCR analyses of genes involved in photosynthesis (*psbA*) disclosed a strong correlation between cyanobacterial activity and water temperature. Metagenomic analyses of experimental microcosm communities revealed that with rising temperatures, blooms of diazotrophs start earlier, and one could expect a shift in the community rather than an intensification of the blooms (Berner et al. 2018).

Generally, metagenomic studies revealed the presence of cyanobacteria from all orders, apart from Stigonematales. The other conclusion is that despite the fairly high cyanobacterial diversity in the Baltic Sea, one can observe a “long tail” phenomenon, with a few taxa dominating and many occurring in small numbers. Rare taxa that can form local blooms are often unseen by HTS projects and can be detected by more classical approaches (Chernova et al. 2019, Overlinge et al. 2021).

Baltic cyanobacteria constitute an unexplored gene pool and, as suggested by some authors, compared to other prokaryotes, their diversity is high (measured using the parameter SESMPD, i.e. Standardized Effect Side of Mean Pairwise Phylogenetic Distance) (Celepli et al. 2017). The interactions between cyanobacteria and associated microbes were studied by Berg et al. (2018). By targeting key gene transcripts, the authors showed that cyanobacterial blooms affect the composition of functional microbial communities.

### In-depth analyses of cultured strains

After the introduction of genetic methods (mainly 16S rRNA sequencing), in the late 20th century, the number of cyanobacterial species, originally identified in the Baltic Sea based on morphological traits, increased significantly. However, in the studies on strains deposited in the culture collections, in-depth and complex analyses incorporated in the polyphasic approach, indicated high intraspecies diversity of these microorganisms (Wulff et al. 2007, Mazur-Marzec et al. 2016, Szubert et al. 2021). Moreover, the genetic diversity of randomly studied benthic cyanobacteria was shown to be even higher than planktic taxa (Surakka et al. 2005, Sihvonen et al. 2007, Halinen et al. 2008). The significance of this genetic and metabolic diversity of Baltic cyanobacteria for the functioning of the ecosystem has yet to be discovered.

To fully understand the diversity of cyanobacteria in the Baltic Sea using environmental DNA analyses, the access to reliable databases containing well-annotated genomes of type taxa is required. Out of 1110/70/263 genomes assembled to levels of scaffold/chromosome/complete genome, respectively, classified to the Cyanobacteria/Melainabacteria group, 7/1/7 belong to Baltic organisms, respectively [Genome List—Genome—NCBI (nih.gov), last accessed on 8th December 2023]. These genomes do not comprehensively represent the Cyanobacteria phylum: 13 represent the Nostocales order, one of the orders Pleurocapsales and Pseudanabaenales. The primary objectives of those genomic studies were (i) to identify gene clusters associated with bioactive metabolite production and, more broadly, to determine the biosynthetic potential of Baltic cyanobacteria, (ii) to verify their taxonomy, (iii) to determine adaptation to variable salinity conditions, and (iv) to identify host–microbiota interactions in holobiont (Vos et al. 2013, Teikari et al. 2018, 2019, Österholm et al. 2020, Ahmed et al. 2021, Bonthond et al. 2021, Dreher et al. 2021, Heinilä et al. 2022). The cyanobacterial genomes found in brackish waters are not well represented in databases, an observation also confirmed by data from Chesapeake Bay (Celepli et al. 2017).

**Figure 3.**
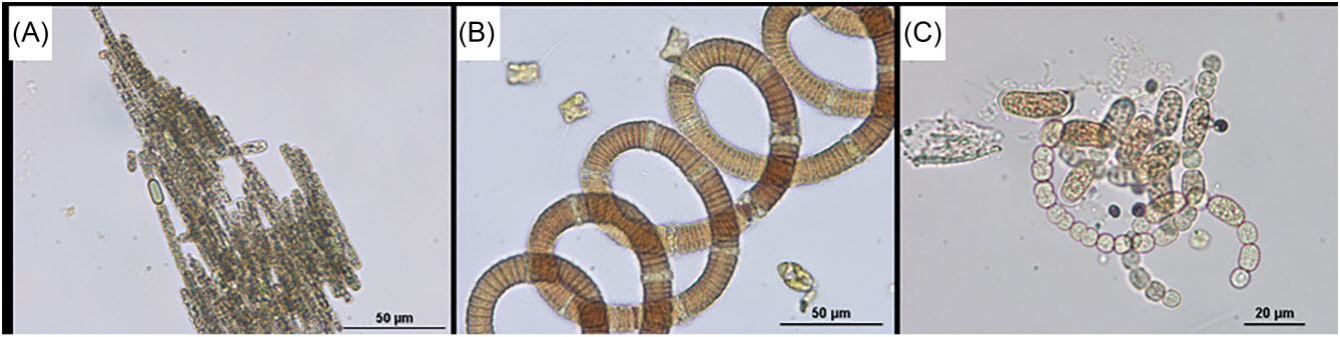
Microphotographs of cyanobacteria from the Baltic Sea. Cells of the following species are shown: *Aphanizomenon flos-aquae* (A), *N. spumigena* (B), and *Dolichospermum lemmermannii* (C). Photographs taken by Justyna Kobos.

Examples of microphotographs of Baltic cyanobacteria are shown in Fig. 3.

## Diatoms in the Baltic Sea

Diatoms (Bacillariophyceae) constitute the largest group of microalgae on Earth. They are present in all marine and terrestrial habitats, where light and humidity are accessible, forming the base of the trophic chain as primary producers (Armbrust 2009). Likewise, diatoms dominate microalgae in terms of biodiversity at the generic and species levels in the Baltic Sea. Published sources on the Baltic Sea diatoms provide numbers ranging from 90 to 143 genera (Pankow 1990) and 633 to 759 species (Hällfors 2004). However, the species diversity, as outlined above in Pankow (1990) and Hällfors (2004), seems to be strongly underscored when compared with reports by Snoeijs and coworkers (Snoeijs 1993, Snoeijs and Vilbaste 1994, Snoeijs and Potapova 1995, Snoeijs and Hajdu 1996, Snoeijs and Balashova 1998), as with 500 species illustrated from the whole Baltic Sea, many species are not listed by the former authors. In a recent publication on diatom assemblages in the Gulf of Gdansk and neighboring waters, Pliński and Witkowski (2020) identified about 900 taxa, a significantly higher number than those listed in the above mentioned reports. The lists of taxa published by Pankow (1990), Hällfors (2004), and Pliński and Witkowski (2020) largely overlap with each other.

The use of molecular tools in assessing the diatom diversity in the Baltic Sea has been launched with the papers by Sarno et al. (2005) and Pniewski et al. (2010) in which a series of common benthic and planktonic brackish/marine water species have been studied with both morphological and molecular methods. Whereas Sarno et al. (2005), using LSU sequences, sorted out the puzzle of species names in the *Skeletonema costatum*-like complex, Pniewski et al. (2010) applied 18S rRNA sequences for reconstructing the phylogeny of the species studied, and 5.8S and ITS2 rRNA as a barcode. Some species studied by Pniewski et al. (2010), and given names of the established species, were isolated again from the western part of the Polish coast. A contribution from Sarno et al. (2005) resulted in establishing *Skeletonema dohrnii* and *S. marinoi* species new to science. Further isolation and research on the Baltic Sea benthic diatom species were continued by Li et al. (2018). That study, based on detailed morphology analysis and three-gene concatenated molecular phylogeny (SSU, *rbcL*, and *psbC*), has revealed a high diversity of benthic diatoms of the Polish Baltic Sea coast, contrarily to the unusual hidden diversity of the splash zone in the Pomeranian Bay At first, Li et al. (2018) sorted out some problematic taxa that are dominant in the Baltic Sea coastal zone, e.g. *Opephora mutabilis, Fragilaria sopotensis*, and *Pseudostaurosira trainorii* and transferred them to *Gedaniella*, a genus new to science established to accommodate the mentioned taxa. Furthermore, *F. sopotensis* turned out to be part of the cell cycle of a diatom from Japanese waters and was named *Fragilaria flavovirens* (Takano 1986). *Fragilaria flavovirens* was transferred to *Gedaniella*, and the species is characterized by a truly worldwide (cosmopolitan) distribution. Li et al. (2018) described species new to science among taxa typical for the brackish waters of the Baltic Sea, like *Gedaniella arenaria*, which in a routine diatom identification with light microscopy and electron microscopy would be included in *Gedaniella guenter-grassii*. It was only the use of molecular markers that allowed the authors to separate the two species. The application of these markers has resulted in some unexpected discoveries in the Baltic Sea diatoms. One of the strains isolated from the sublittoral waters of Sopot was positioned in the phylogenetic tree in a small clade with *Plagiostriata goreensis*, a formerly monotypic diatom genus known thus far only from its type habitat in the Goree Island in Senegal, tropical East Atlantic (Sato et al. 2009). *Plagiostriata baltica* is the second species in the genus and the first occurring in a brackish-water habitat (Li et al. 2018).

The biodiversity of the Baltic Sea diatom assemblages is nearly exclusively based on the analyses performed with purified material in light and electron microscopic studies. Studies based on clonal cultures of the Baltic Sea strains are still rare and involve the most common planktonic species, *S. marinoi* (Godhe and Härnström 2010, Pinseel et al. 2022), or a few dozen strains isolated from littoral benthic habitats of the Southern Baltic Sea coast (Pniewski et al. 2010, Li et al. 2018, Prelle et al. 2022). Likewise, metabarcoding is becoming a standard tool for phytoplankton biodiversity assessment for both freshwater and marine ecosystems worldwide (Apothéloz-Perret-Gentil et al. 2017, Pérez-Burillo et al. 2021, 2022, Bilbao et al. 2023, Kaleli et al. 2023), yet for the Baltic Sea such approach remains uncommon. Baltic Sea diatom-focused metabarcoding occurs in literature only as a part of larger phytoplankton studies (Gran-Stadniczeñko et al. 2019, Andersson et al. 2023), where authors performed analyses of phytoplankton using both microscopy and metabarcoding. The explanation for the issue of low interest in the metabarcoding study may be related to the excellent biomonitoring organized in the Baltic Sea through the HELCOM, where the experts on all classes of microalgae analyse phytoplankton and phytobenthos of particular regions and the results are published regularly (Wasmund and Uhlig 2003, Ojaveer et al. 2010, Olofsson et al. 2020, Fridolfsson et al. 2023).

The use of cultured strains allows various experiments on the Baltic Sea diatoms to be performed. These include studies on growth rate dependence on salinity, temperature, and nutrients as well as on the dependence on bioactive compounds synthesized by the diatom strains (like lipids and fatty acids). One of the critical questions in the case of the Baltic Sea planktonic strains represented by *S. marinoi* is the adaptation of the species to the salinity gradient in the Baltic Sea basin. Pinseel et al. (2022) studied the transcriptomic response of the Baltic Sea *S. marinoi* strains isolated from an environment close to marine conditions and further inside the basin from low salinities. The results of that study underlined the environmental factors, including salinity, temperature, and nutrient uptake, as the cause of the biological variation. A similar experiment was also carried out by Prelle et al. (2022), but included only the response of benthic species representing the genus *Planothidium* from the coastal wetlands in the eastern part of the German coast. In a series of ecophysiological experiments, the authors noted the presence of two different ecotypes of *Planothidium* sp., which were identical in terms of their *rbcL* sequences. Interestingly, regarding salinity response, the two *Planothidium* sp. strains differed significantly, with one strain isolated from the brackish-water reaching its maximal growth rate in salinity of 15 PSU and the strain isolated from freshwater in the lower salinity range.

**Figure 4.**
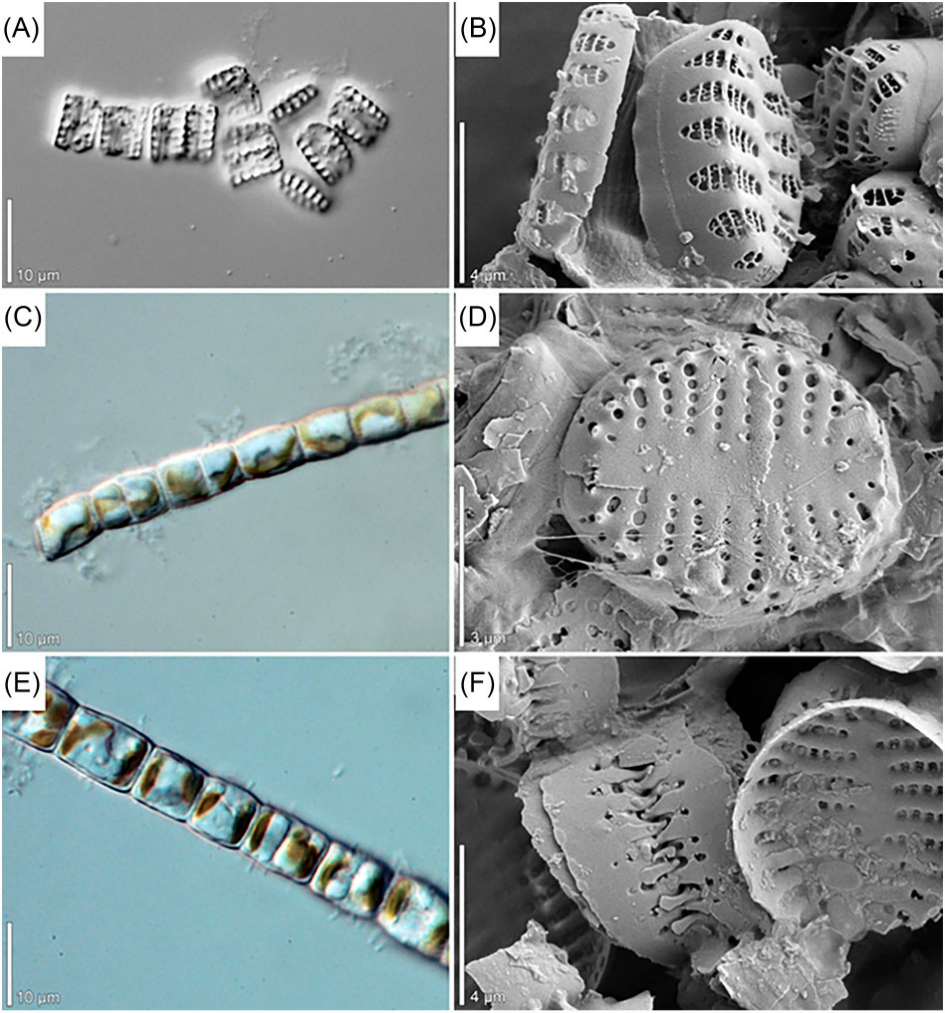
Microphotographs of diatoms from the Baltic Sea. Cells of the following species are shown in images obtained using light (A, C, and E) and electron (B, D, and F) microscopic techniques: *Gedaniella mutabilis* (A and B), *P. trainorii* (C and D), and *Gedaniella flavoviren* (E and F). Photographs taken by Przemysław Dąbek.

In summary, although diatoms are apparently the largest group of microalgae in the Baltic Sea, the scarcity of molecular data regarding this group of microorganisms is a serious limitation in performing a comprehensive analysis of the biodiversity of this group. Examples of microphotographs of Baltic diatoms are shown in Fig. 4.

## Dinoflagellates in the Baltic Sea

Dinoflagellates (Dinophyceae FE Fritsch) is a diverse group of mainly unicellular protists, some forming chains or colonies. In the Baltic Sea (including the Kattegat), 139 morphological species are monitored using a light microscopy-based method (Olenina et al. 2006). In the International Code of Nomenclature for algae, fungi, and plants (ICN), dinoflagellates are mainly found in the class Dinophyceae as reflected in Algae Base (Guiry and Guiry 2023). The International Code of Zoological Nomenclature (ICZN) also includes dinoflagellates. However, the ICZN nomenclature is not used in the current text. The application of phylogenomics shows that dinoflagellates form a subgroup within the *Alveolata* clade, which is a part of the larger group TSAR, that includes *Telonemia, Stramenopiles, Alveolata*, and *Rhizaria* (Burki et al. 2020). There are phototrophic, heterotrophic, mixotrophic, and parasitic dinoflagellates. In this case, mixotrophy is defined as feeding on other microorganisms. Feeding strategies are diverse and include catching prey using peduncle (Spero 1982, Hansen 1991), mucus traps (Blossom et al. 2012, Papiol et al. 2016) and engulfment (Skovgaard 1996). Many dinoflagellates are functional phytoplankton or microalgae since they have chloroplasts. Most have permanent chloroplasts with peridinin as a pigment, distinguishing them from other phytoplankton groups (Schnepf and Elbrächter 1999, Zapata et al. 2012). They can also have acquired chloroplasts from several different photosynthetic microorganisms, including diatoms (Bacillariophyceae), e.g. *Peridinium balticum* (Chesnick et al. 1997), haptophytes (Haptophyta, Prymnesiophyceae), e.g. *Karenia mikimotoi* (Zapata et al. 2012), cryptophytes (Cryptophyceae), e.g. *Dinophysis* (Rial et al. 2013), and chlorophytes (Chlorophyta), e.g. *Lepidodinium chlorophorum/viridae* (Zapata et al. 2012).

Bioluminescence, which is common among dinoflagellates, has been shown to reduce grazing by copepods (Prevett et al. 2019). In the Baltic Sea, a bioluminescence from *Alexandrium ostenfeldii* (Paulsen) Balech and Tangen was reported from the Stockholm Archipelago (Karlson et al. 2021), Åland Archipelago, and Puck Bay (Kremp et al. 2009, Hakanen et al. 2012). Many dinoflagellates have resting stages called cysts that improve survival and act as seed populations. The distribution of the cysts has been investigated in parts of the Baltic Sea (Ellagaard et al. 1994, Nehring 1994, 1997).

Certain dinoflagellates are harmful in some way. There are 106 species of dinoflagellates listed in the IOC-UNESCO Taxonomic Reference List of Harmful Micro Algae (Lundholm et al. 2023). Eighteen of these have been observed in Northern European coastal seas (Karlson et al. 2021). Examples from the Baltic Sea include *Alexandrium* spp., producers of paralytic shellfish toxins, *Dinophysis* spp., producers of diarrhetic shellfish toxins, and *Karlodinium veneficum* causing fish mortalities (Karlson et al. 2021).

Dinoflagellate abundance is largely controlled by bottom-up factors such as light and nutrient availability, but also by competition and top-down control. Grazing by zooplankton, and in some cases by other dinoflagellates (Rodriguez et al. 2014), also controls dinoflagellate abundance. Moreover, parasitism (exemplified by *Parvilucifera* spp.) affects the dinoflagellates (Alacid et al. 2020).

Planktonic dinoflagellates are inspected as part of the Baltic Sea’s phytoplankton international and regional monitoring programs. Most international programs are coordinated through HELCOM, and methods are largely standardized through the HELCOM COMBINE manual (HELCOM 2017). Samples are taken near the surface, usually at depths of 0–10 m, and the frequency is often monthly. The number of stations sampled varies by country, for example, in the Swedish program, about 10 stations are sampled monthly. The current sampling programs do not resolve natural variability satisfactorily. Samples are analysed by light microscopy using the sedimentation chamber method (Utermöhl 1958, Edler and Elbrächter 2010). The taxa are most often defined at the species or genus level. In addition, the cell volume, and the trophic type (phototrophic, heterotrophic, mixotrophic, or unknown) are determined based on a checklist updated yearly (Olenina et al. 2006). The current version of the document is available on the following web page: https://www.ices.dk/data/Documents/ENV/PEG_BVOL.zip. Biomass in carbon is calculated based on observed cell volumes (Menden-Deuer and Lessard 2000).

Novel methods for dinoflagellate analysis are available and are being applied in the Baltic Sea. Here, automated analyses using imaging in-flow systems and molecular methods are being addressed. DNA metabarcoding is used to investigate the distribution and diversity of dinoflagellates and other plankton (Hu et al. 2016, Sildever et al. 2021). Benthic heterotrophic protist communities were investigated in the Southern Baltic Sea (Sachs et al. 2023). The target gene is mainly 18S rRNA, but the ITS and 28S regions are also used (Latz et al. 2022). When using both the V3–V4 and the V9 regions of 18S, it was noted that the different regions resolved dinoflagellate species differently (Gaonkar and Campbell 2023). Standardized DNA extraction, sequencing, and bioinformatic protocols have been recommended (Andersson et al. 2022, Jerney et al. 2022, 2023). DNA is sequenced using Illumina NextSeq, MiSeq, PacBio, or Oxford Nanopore platforms. A bioinformatics processing pipeline such as DADA2 (Callahan et al. 2016) is applied, and the resulting ASVs are compared to reference databases, e.g. the Protist Reference Database 2 (PR^2^) (Guillou et al. 2013). The results are often presented as relative abundance, i.e. the number of gene copies of a taxon divided by the total number of gene copies for all taxa observed in a sample. This is useful for showing diversity, as more taxa are observed using metabarcoding compared to microscopy. The main reason is likely that taxa with few morphological features are not distinguished using light microscopy. Another reason may be that a larger sample volume is analysed using metabarcoding, often ∼500 ml, compared to ∼20 ml for microscopy. DNA metabarcoding reveals a larger diversity of dinoflagellates compared to microscopy. This likely results from the fact that taxa with similar morphology can be differentiated using biogenomics, especially small dinoflagellates. Moreover, parasitic dinoflagellates may be revealed using metabarcoding (Käse et al. 2021). A disadvantage of metabarcoding is that the results are not directly comparable to cell abundance or biomass estimated by microscopy. Another problem is that many dinoflagellate taxa are currently missing in the reference databases. In addition, the variability in the 18S seems to be too low to resolve many dinoflagellate taxa. Therefore, sequencing longer stretches of the genome is recommended.

As mentioned earlier, qPCR is a molecular method helpful for analyses of the abundance of dinoflagellates and other planktonic organisms (Dittami et al. 2013, Ruvindy et al. 2018). Processing samples is quick, but only a few taxa can be analysed simultaneously using this method. Therefore, qPCR (or ddPCR) is applicable for observing harmful dinoflagellates but not for exploring the whole phytoplankton community. As was also already mentioned, a methodological breakthrough was the introduction of the IFCB (Olson and Sosik 2007, Sosik and Olson 2007). IFCB has been widely used for observations of dinoflagellates (Campbell et al. 2010, 2013, Brosnahan et al. 2015). There are other imaging in-flow instruments available for dinoflagellates observations, e.g. the CytoSense (Cytobuoy b.v., the Netherlands). A general requirement for automated imaging in-flow analyses of plankton is that images of plankton identified by phytoplankton specialists are needed to train the automated algorithms.

The following conclusions can be drawn: (i) automated imaging-in flow systems are valuable tools for increasing the sampling frequency of dinoflagellates and other phytoplankton; libraries of reference images are needed to improve automated identification of taxa, (ii) DNA metabarcoding is helpful in investigating dinoflagellate diversity and distribution; metabarcoding reveals a higher diversity than light microscopy; for dinoflagellates, many taxa are missing in reference databases; 18S rRNA may have too low variability to resolve species; longer sequences, including also ITS and 28S regions may be needed, (iii) qPCR (and ddPCR) is useful for quantitative analysis of a limited number of dinoflagellate species, thus, it is recommended for analysing selected harmful taxa.

**Figure 5.**
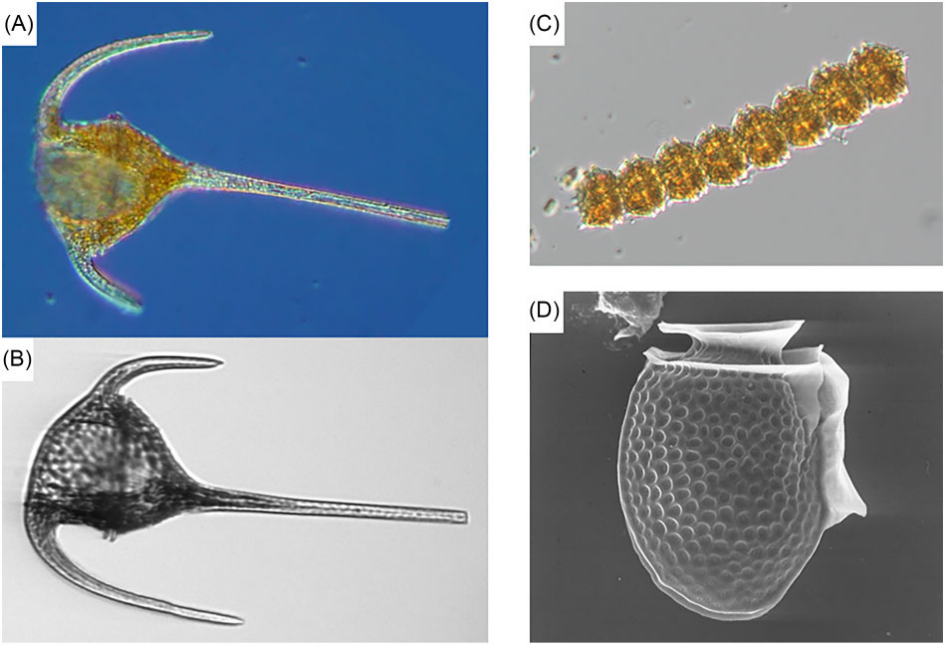
Microphotographs of dinoflagellates from the Baltic Sea. Cells of the following species are shown in images obtained using light microscopy (A and C), Imaging Flow Cytobot (B), and electron microscopy (D): *Tripos mulleri* (A and B), *Peridiniella catenata* (C), and *Dinophyis acuminata* (D). Photographs taken by Maria Karlberg and Bengt Karlson.

Examples of microphotographs of Baltic dinoflagellates are shown in Fig. 5.

## Other protists in the Baltic Sea

Metagenomic studies of nanoplanktonic protists and ciliates lag behind those of prokaryotes. The main reason is that assembling good-quality eukaryotic MAGs is challenging due to the complexity of their genomes. So far, most studies that use environmental DNA to analyse protist diversity are based on HTS of barcoded amplicons of the 18S rRNA gene. Here, we review those few that focused on the Baltic Sea.

Substantial changes in microbial eukaryotic communities along the horizontal salinity gradients are well-described for microphytoplankton (Ojaveer et al. 2010, Wasmund et al. 2017). Maximum diversity is observed at salinity 5–8 PSU, called horohalinicum (Telesh et al. 2011), where marine and freshwater protistan species can live (Telesh et al. 2013, 2015). This large-scale alpha-diversity pattern was confirmed for smaller protists with HTS studies (Hu et al. 2016, Filker et al. 2019). Two salinity thresholds within horohalinicum were identified: at around 10 PSU, at which dominance of dictyochophytes shifts to dinophytes (Filker et al. 2019), and at about 6 PSU, where dinophytes are replaced by ochrophytes (Hu et al. 2016). These studies also indicated that ciliates (Ciliophora) rise at salinity below 9.5 PSU (Hu et al. 2016, Filker et al. 2019). A similar pattern was observed at a local scale along the gradient from 0 to 7 PSU in the estuary of the Vistula River (Piwosz et al. 2018). It was observed that protist community changes along vertical salinity gradients were less pronounced in spring when water was oxygenated almost to the bottom (Filker et al. 2019). This is likely very different in summer, when waters below the halocline become anoxic and even sulfidic (Anderson et al. 2013). For instance, it is well-known that ciliates are particularly important in oxygen-deficient zones (Stock et al. 2009), and Baltic anoxic zones are apparent targets of future studies using HTS methods. In the Gotland Deep, the application of older molecular methods revealed higher phylotype diversity than previously detected during microscopy- and cultivation-based studies (Stock et al. 2009, Weber et al. 2014).

The main advantage of the HTS studies is a deeper insight into protistan diversity. At the same time, decadal-long studies of microphytoplankton in the whole Baltic produced a list of some 2700 species, estimates by HTS methods ~2000 OTUs/ASVs from a single study (Hu et al. 2016, Piwosz et al. 2018, Filker et al. 2019). The list of newly found protists includes, among others, phagotrophic Cercozoa (other than Ebria), Katablepharidophyta, Telonemia, Picozoa, MAST lineages (such as MAST-1, 2, 3, 4, and 6), Acantharea, and aplastidic cryptophytes from the CRY1 lineage, as well as parasitic Syndiniales and Oomycetes (Hu et al. 2016, Piwosz et al. 2016, 2018, Eigemann and Schulz-Vogt 2019, Filker et al. 2019, Piwosz 2019). However, the most commonly used primer sets for the V4 and V9 regions of the 18S rRNA gene poorly cover Excavata (Amaral-Zettler et al. 2009, Stoeck et al. 2010, Balzano et al. 2015). Recently, a new primer pair for the V6–V8 region with good coverage for all eukaryotic supergroups has been designed and tested in the Baltic Sea (Latz et al. 2022). However, their application in the Bothnian Bay did not show Excavates to contribute to microbial eukaryotic communities substantially (Eriksson et al. 2022, 2023).

The diversity of nanosized protists and ciliates is even more understudied in nonpelagic environments of the Baltic Sea. Benthic protistan communities were shown to be dominated by dinoflagellates and diatoms, with minor contributions from ciliates and perkinsenids (Salonen et al. 2018). In contrast, ciliates (Ciliophora), followed by Dinoflagellata and Cercozoa, were the most frequent protists in the sediment surface layer (Sachs et al. 2023). Finally, floating microplastic particles became an important substrate for protists (Kettner et al. 2019). Although the community composition did not differ between polyethylene, polystyrene, and wood particles, potentially harmful dinoflagellate *Pfiesteria* was substantially enriched on microplastic particles, indicating a future threat to other organisms as these particles accumulated.

The knowledge gaps in protistan communities are evident. A few snapshots and local studies do not provide an exhaustive overview of their diversity, even in the best-investigated euphotic layers of the Baltic Sea. Communities living in deep waters, sediments, and submerged living and nonliving surfaces still await description. The amplicon-based studies, most commonly used for protistan communities, do not provide quantitative information on the abundance of specific lineages (Piwosz et al. 2020). Thus, they should be treated predominantly as qualitative data. Obviously, this is not enough to understand how protists function in aquatic ecosystems (Piwosz et al. 2021). Hopefully, the advent of meta-omics approaches to studying protists will open a new chapter in their ecology (Delmont et al. 2022). Before these approaches reach the accuracy level currently available for prokaryotes, long amplicons covering the whole rRNA operon may help to bridge the gap (Latz et al. 2022). Moreover, the morphological diversity of some protists, such as ciliates or dinoflagellates, is much greater than that of prokaryotes. Thus, it is recommended to conduct studies providing both molecular and morphological data simultaneously (Saldarriaga et al. 2004, Weber et al. 2014). Such an approach offers molecular sequences for taxonomically identified organisms.

**Figure 6.**
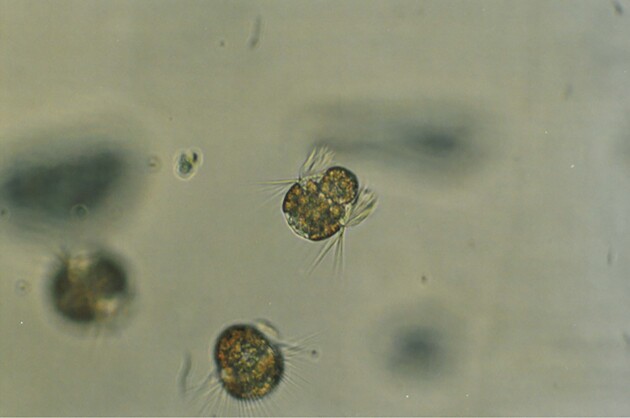
Microphotograph of a ciliate from the Baltic Sea. Cells of *Mesodinium major* are shown in the microphotograph. Photograph taken by Krzysztof Rychert.

A microphotograph of an example of a Baltic ciliate, *Mesodinium major* Garcia-Cuetos, Moestrup & Hansen, 2012 (the species detached from *Mesodinium rubrum*; Lohmann 1908), is shown in Fig. 6.

## Fungi in the Baltic Sea

Compared to bacteria and some groups of algae, fungi are an understudied microorganism group, and vast parts of the Baltic Sea are still awaiting mycological exploration (Tibell et al. 2020). Fungi comprise substantial quantities of biomass in the marine realm (Hassett et al. 2019), but their activity must still be fully represented in marine ecosystem models (Vass et al. 2022). In the Baltic Sea, fungi were discovered in the late 19th century, with more detailed investigations undertaken during the 20th century (Tibel et al. 2020). The main factors controlling fungal diversity in coastal ecosystems include salinity, DO, and nutrient conditions (Hassett et al. 2019, Rojas-Jimenez et al. 2019). Salinity is one of the shaping parameters of pelagic fungal community composition in the Baltic Sea, where significant differences are observed above and below a critical value of 8 PSU (Rojas-Jimenez et al. 2019). However, the benthic fungal diversity of the Baltic Sea is shaped by water depth, salinity, and sediment C and N availability (Lobo et al. 2024).

The fungal diversity of the Baltic Sea has not yet been fully revealed. It has one of the highest richness of explored marine areas worldwide (Hassett et al. 2019). Recent investigations based on HTS data revealed 319–345 OTU of fungal sequences along the salinity gradient (3–34 PSU) (Rojas-Jimenez et al. 2019) or 493 fungal taxa in the subarctic zone of the Baltic Sea (Vass et al. 2022). However, based on fungi morphological features supported by DNA diagnostic, only 77 true marine fungi species belonging to filamentous ascomycetes and basidiomycetes were found on driftwood, live and dead macroalgae, and shore plants (Tibell et al. 2020). There are ongoing debates on which fungi present in seas should be considered typical marine fungi. As has been agreed lately (Pang et al. 2016), a marine fungus is “any fungus that is recovered repeatedly from marine habitats because (i) it can grow and/or sporulate in marine environments; (ii) it forms symbiotic relationships with other marine organisms, or (iii) it is shown to adapt and evolve at the genetic level or be metabolically active in marine environments.” Since the use of the “marine fungi” concept is questionable in the Baltic Sea, which is considered brackish water, Tibell et al. (2020) assigned to marine fungi only the species that were previously reported from oceanic waters (apart from the Baltic Sea). Two of them belonged to Basidiomycota (*Digitatispora marina* and *Leucosporidium scottii*), while others belonged to the Ascomycota, mostly from Sordariomycetes and Dothideomycetes classes. Most of those species have their main distribution in temperate waters of the Atlantic Ocean. However, 13 species were found for the first time in the Baltic Sea. Several, like *Halojulella avicenniae, Leptosphaeria australis, Setoseptoria phragmitis*, and *Trichocladium melhae*, were previously found only in faraway tropical areas.

The studies based on HTS revealed that Rozellomycota (syn. Cryptomycota) and Chytridiomycota are the dominant representatives of the pelagic community of the Baltic Sea (Rojas-Jimenez et al. 2019, Vass et al. 2022, Hassett et al. 2019). The dominance of Rozellomycota reflects the patterns observed in lacustrine ecosystems (Rojas-Jimenez et al. 2017). This is an understudied phylum of intracellular parasites of zoosporic fungi and Oomycota that grow as naked protoplasts within their hosts (Ilicic and Grossart 2022). Recently, 25 associations found between fungal and algal OTUs suggest potential host–parasite and/or saprotroph links, supporting a Cryptomycota-based mycoloop pathway in the Baltic Sea (Vass et al. 2022). Another dominant mycoplankton and mycobenthos group in the Baltic Sea, Chytridiomycota, are facultative or obligate parasites of macroalgae, diatoms, and dinoflagellates. Their dominance in benthic environments is partly explained by the sedimentation of phytoplankton blooms (Lobo et al. 2024). Even though they are relatively highly abundant, much of the diversity known within these groups is almost entirely based on environmental sequencing data (Karpov et al. 2021). Chytridiomycota is an understudied clade of marine fungi that might have greater ecological relevance than is currently recognized. Partly, this is due to the lack of annotated reference sequences of marine chytrids in DNA databases, making it difficult to determine the identity, ecological role (parasitic or saprophytic), and host interactions (Fernández‐Valero et al. 2022, Ilicic and Grossart 2022).

Few new species from the Baltic Sea are described by combining light microscopical observations, ultrastructure, and molecular phylogenetic analysis, for example, the parasitic chytridiomycete—*Ericiomyces syringoforeus* gen. et sp. nov. (Karpov et al. 2021) or *Paradinomyces triforaminorum* gen. et sp. nov. (Reñé et al. 2022) co-occurring with other parasitoids during *Kryptoperidinium foliaceum* blooms. Therefore, studies that combine cultivation, host range determinations, single-cell PCR techniques, and genome sequencing are needed to understand the structure and function of the chytrid community, the parasitic and competitive strategies, as well as the ecological or temporal niches of chytrids (Fernández‐Valero et al. 2022, Van den Wyngaert et al. 2022). Parasitic fungi affect microbial interactions through several mechanisms: (i) transferring photosynthetic carbon to infecting fungi, (ii) stimulating bacterial colonization on phytoplankton cells, and (iii) altering the community composition of bacteria and their acquisition of photosynthetic carbon (Klawonn et al. 2021). Fungal microparasites can affect microorganisms-related carbon flow at the base of aquatic food webs and should be considered essential members of plankton communities. Previous studies investigating fungal interactions have mainly been restricted to studies in laboratory settings, not providing information about how these interactions impact the distribution of mycoplankton communities in nature (Vass et al. 2022). The contribution of biotic associations to fungal metacommunity assembly is essential to improve predictions of species distributions in aquatic ecosystems. Identifying biotic relationships that affect the distributions of members of mycoplankton could be helpful in plankton ecology through habitat management to promote species that control algal blooms and facilitate nutrient transfer to upper trophic levels (Vass et al. 2022).

The pattern observed across culture-based studies of host-associated fungi shows that ubiquitous fungi belonging to *Penicillium, Aspergillus*, and other fungi found in the terrestrial environment are the dominant members of such communities. These fungi are highly adaptable and survive in various extreme conditions. The ocean is considered a sink for terrestrial-sourced fungi, of which a large fraction can reproduce in marine conditions due to evolutionary adaptations. Thus, coastal areas and enclosed seas, such as the Baltic Sea, with significant terrestrial influence, are considered biological hotspots for marine fungal diversity (Hassett et al. 2019).

The host-associated fungi have been gaining more attention recently due to their potential to produce metabolites. Altogether, 55 fungi were isolated and identified from the Baltic seaweed *F. vesiculosus* (Fan et al. 2019), with a significant part consisting of endophytes and only a few epiphytes of *Gibellulopsis, Phoma*, and *Penicillium* genera. Based on metagenomic analysis, Ascomycota was the most abundant phylum on different species of *Fucus* surfaces from the Baltic Sea environment (Oppong-Danquah et al. 2023). On *F. vesiculosus*, the yeast *Candida* was a significantly abundant ascomycete genus, which might contribute to microfouling and consume algal exudates, while on *F. distichus* subsp. *evanescens* was *Mucor*, whose major secondary metabolite is tyrosol, known to display antibiofilm activities (Oppong-Danquah et al. 2023). As many as 13 fungal strains, dominated by *Penicillium* species (10 strains), were isolated from the leaf and the root rhizosphere of the Baltic *Zostera marina* (Petersen et al. 2019). In total, 22 strains of fungi were for the first time isolated from the tunic of *C. intestinalis* collected from the North and Baltic Seas (14 strains) (Utermann et al. 2020). Much lower diversity was identified in association with flatfish European plaice (*P. platessa*) (six isolated species in total from the stomach, intestine digest gut epithelium) (Ghotbi et al. 2022).

Fungi are much less abundant in association with macroorganisms than bacteria but produce interesting and previously unknown secondary metabolites. Still, there are only a few studies on that aspect. From *F. vesiculosus* found in the Baltic Sea, isolated *Pyrenochaetopsis* sp. yielded newly identified pyrenosetins A–D, phomasetin, and wakodecalines A and B (Fan et al. 2020). The presence of many potentially new decalinoylspirotetramic acid derivatives was also indicated (Fan et al. 2022). *Pyrenochaetopsis* sp. and related pyrenosetins would be of interest for further research to understand their biosynthesis, particularly their true chemical diversity and bioactivity (Fan et al. 2022). The metabolome of the tunic-associated fungus extracts indicated a high chemical diversity of compounds putatively assigned to alkaloids, lipids, peptides, polyketides, and terpenoids. Many detected metabolites could not be annotated to known natural products and are classified as newly discovered (Utterman et al. 2020). From a plaice intestine-epithelium-derived fungus *Aureobasidium pullulans*, PI9-F had remarkable chemical diversity and was active against the fish pathogen *Lactococcus garvieae*, methicillin-resistant *Staphylococcus aureus*, and *Enterococcus faecium* (Ghotbi et al. 2022). While we have more knowledge about host-associated fungi diversity and their metabolites in the Baltic Sea (Petersen et al. 2019, Utermann et al. 2020, Ghotbi et al. 2022), there is still very little information about how they modulate the host–microbe interactions, thereby possibly affecting the health of the host.

Moreover, not only host-associated fungi are metabolically diverse. Dendrodolide N and E (compounds from the group of macrolides), spiciferinone (azaphilone chromophore), and the new analogue 8a-hydroxy-spiciferinone, and cephalochromin were isolated from fungi *Plenodomus influorescens* and *Pyrenochaeta nobilis*, obtained from marine sediment of Baltic Sea (Oppong-Danquah et al. 2020). Thus, fungi derived from the Baltic Sea environment are the untapped source of newly discovered marine natural products with pharmacological relevance, especially for antimicrobial and cytotoxic activities (Utterman et al. 2020, Ghotbi et al. 2022).

Fungi are a phylogenetically diverse group, and only around ∼50% of the known marine fungal species have an available DNA locus in public databases, and the Baltic Sea is not an exception (Hassett et al. 2019). Expanding the collection of reference loci/genes and genomes will be fundamental in understanding the ecology of marine fungi to support the HTS data (Tibell et al. 2020). Detailed taxonomic information was lacking for most Cryptomycota OTUs (88.2%) investigated north of the Baltic Sea (Vass et al. 2022). HTS offers ways to understand marine fungal diversity patterns at higher taxonomic resolutions (phylum, family, or order level) (Hassett et al. 2019, Rojas-Jimenez et al. 2019). Studies on fungal DNA metabarcoding use markers depending on the taxonomic group of interest and the desired resolution. Long-read metabarcoding covering 18S-ITS1-5.8S-ITS2-28S rRNA (Wurzbacher et al. 2019) has been used recently in the Baltic Sea to overcome the phylogenetic diversity of fungi (Vass et al. 2022), thus enabling the use of different rRNA gene reference databases and significantly improving taxonomic classification over a single gene marker approach (Ilcic and Grossart 2022).

Although there is increasing knowledge about fungal diversity, we still lack information about their biomass, which is essential for understanding the functional role of fungi in the Baltic Sea. Fungi biomass based on 5.8S rRNA genes was quantified by qPCR in oil-polluted sediments of the Baltic Sea (Mietinnen et al. 2019). Based on the results, the abundance of fungi in contaminated water and sediment samples was lower than that of bacteria and archaea. Moreover, the presence of oil in the environment significantly lowered the abundance of fungi under experimental conditions, but it did not affect the diversity of fungi in water and sediment (Yan et al. 2020). For the detection and quantification of fungal parasites, a dual use of fungal cell wall markers, calcofluor white (CFW) and wheat germ agglutinin (WGA), is suggested. It allows the detection and quantification of multiple, primarily undescribed chytrids infecting phytoplankton in freshwater and the Baltic Sea environment (Klawonn et al. 2023).

A significant group of fungi are saprophytes; thus, the role of fungi in degrading anthropogenic pollutants as the source of carbon accumulated in the Baltic Sea might be crucial. Most recent studies are related to their role in oil degradation (Mietinnen et al. 2019, Yan et al. 2020). Fungi usually are primary decomposers of high-molecular weight hydrocarbons via secreted extracellular enzymes supported by the enzymatic activity of bacteria. The first in-depth report about fungal diversity on another anthropogenic pollutant, microplastic, in different aquatic ecosystems, including the Baltic Sea, revealed that fungal communities on microplastic differed from those in water and on wood as a natural substrate. As parasites, pathogens, symbionts or saprobes, and fungi could substantially influence microplastic biofilm community dynamics and cycling processes (Kettner et al. 2017).

Currently, fungi are gaining more attention due to concerns for human health (Lass-Florl et al. 2022). Terrestrial–coastal interfaces are important for recreational activities where beachgoers might be exposed to potential pathogens. Fungi are missing from water and sand health protection regulatory parameters. Still, it is suggested that in addition to sand, the water should be monitored for potentially pathogenic fungi, at least *Candida albicans* and dermatophytes (Brandão et al. 2021). Based on the sampling data and spatial distribution modeling approach, due to strong eutrophication conditions (chl-a), the probability of occurrence of *Candida* spp. in the Baltic Sea waters is higher compared to less eutrophic coastal waters (Cogliati et al. 2023). Still, further research is needed to better characterize the fungal water contaminants, allowing a better assessment of possible future regulatory parameters.

**Figure 7.**
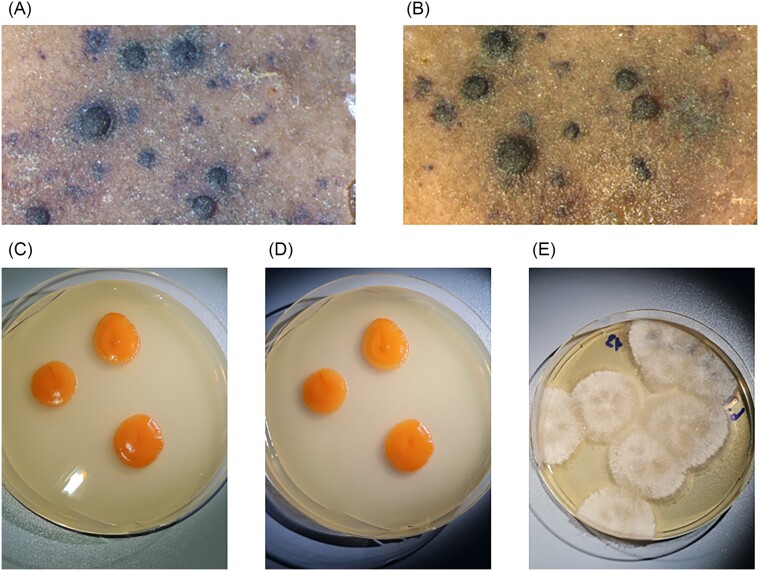
Photographs of fungi from the Baltic Sea. *Ascomycota perithecium* on *F. vesiculosus* blade (A and B), and colonies of *Rhodotorula* sp. (C and D) and *Fusarium* sp. (E) are shown. Photographs taken by Marija Kataržytė.

Summarizing, the recent focus in the Baltic Sea related to fungi is on revealing their diversity based on morphological features supported by DNA-based diagnostics (ITS locus or 18S rRNA) (Tibell et al. 2020) and HTS data (Hassett et al. 2019), investigating the effects of environmental conditions (Rojas-Jimenez et al. 2019) and biotic associations, contributing to the assembly of mycoplankton (Vass et al. 2022) or mycobiome (Ghotbi et al. 2022). Marine fungi are receiving increased attention concerning human health, both as potential pathogens of humans exposed to water (Brandão et al. 2021, Cogliati et al. 2023) and for their bioactive metabolites (Fan et al. 2020, 2022). Still, it is important to highlight that the current fungal biodiversity is at risk of species shift or decline with predicted changes in salinity due to climate change and intensified eutrophication (Lobo et al. 2024). Examples of (micro)photographs of Baltic fungi are shown in Fig. 7.

## Viruses in the Baltic Sea

Reports on viruses occurring in the Baltic Sea are relatively scarce. Nevertheless, from the literature data discussed below, it appears that bacteriophages (or shortly—phages, i.e. viruses infecting bacteria and other prokaryotic hosts) are the most abundant viruses in the Baltic Sea. This is perhaps not surprising, as bacteriophages are considered the most abundant biological entities on Earth, playing enormously important ecological functions (Batinovic et al. 2019, Węgrzyn 2022). Therefore, we divided this chapter into two parts, devoted to bacteriophages and eukaryotic viruses (i.e. those infecting eukaryotic hosts).

### Bacteriophages in the Baltic Sea

The predominance of bacteriophages among all viruses found in oceanic and marine waters is not restricted to the Baltic Sea, but it is a common feature of viral populations in various habitats (Angly et al. 2006, Williamson et al. 2012, Mizuno et al. 2016, Zheng et al. 2021a). In earlier reports on the environmental significance of bacteriophages, these viruses were mistakenly classified as predators (Angly et al. 2006); however, following a long-lasting controversy in the literature, it has been concluded that they are obligatory parasites and should be classified as such (Węgrzyn 2022). Irrespective of the classification, it is evident that bacteriophages, through infecting and either killing their hosts (during lytic development) or changing their properties (in the lysogenic state), effectively affect populations of bacteria and other prokaryotes in marine environments, as discussed below.

The abundance, diversity, and distribution of bacteriophages have been studied using both standard culture-dependent methods and culture-independent novel methods, such as metagenomics and proteomics (Holmfeldt et al. 2014, Howard-Varona et al. 2017, Nilsson et al. 2022), also using advanced bioinformatic tools, like CheckV (Nayfach et al. 2021). Baltic Sea phage abundance has been evaluated using FC (Holmfeldt et al. 2010, Köstner et al. 2019), fluorescent microscopy (Weinbauer et al. 2003, Šulčius et al. 2011, Cai et al. 2019), and electron microscopy (Šulčius et al. 2011, Jakubowska-Deredas et al. 2012). The latter enabled morphological characteristics of phage virions (Jakubowska-Deredas et al. 2012, Cai et al. 2019). Phage diversity has been studied by many authors who focused on phage isolation and characterization, involving virion morphology studies, virus–host interaction studies, including host range, adsorption, and burst size determination (Wolf et al. 2004, Jenkins and Hayes 2006, Luhtanen et al. 2014, Senčilo et al. 2015, Šulčius et al. 2015, Nilsson et al. 2019, 2020, Castillo et al. 2021, Hoetzinger et al. 2021, Stante et al. 2023). In those studies, novel, isolation-independent methods were treated as complementary methods in exploring viral diversity. For example, they were employed to map the environmental metagenomic reads to the sequenced genomes of phages or host bacteria (Senčilo et al. 2015, Nilsson et al. 2019, 2020, Šulčius et al. 2019).

In terms of morphology, three-tailed virus-like morphotypes were identified in the Baltic Sea, namely those with long noncontractile tails, long contractile tails, and short tails (members of the former families *Siphoviridae, Myoviridae*, and *Podoviridae*, respectively), as well as those forming icosahedral particles without tails, belonging to divergent subfamilies within *Microviridae* (Cai et al. 2019, Holmfeldt et al. 2013). Phages of siphoviral morphology seem to dominate in the marine environment. The presence of siphoviruses with extremely long tails has been observed in the sediments obtained from the Gulf of Gdansk (Jakubowska-Deredas et al. 2012). A large number of filamentous, spherical, encapsulated, rod-shaped, and spindle-shaped virus-like particles (VLPs), similar to nuclear-cytoplasmic large DNA viruses, rarely found in a water column, were observed in the deeper sediment. Importantly, intact VLPs were observed in deep subseafloor prokaryotic cells for the first time, thus demonstrating the *in situ* assembly of viral particles in the hosts (Cai et al. 2019).

The isolation studies, although restrictive since a high number of viruses and/or their hosts cannot be propagated under laboratory conditions, enabled the total genome sequencing of about 280 bacteriophages obtained across the Baltic Sea, and delivered important information on phage genomics and phage–host interactions. Most of the phages with sequenced genomes belong to the class of tailed viruses (Caudoviricetes). Their genome (dsDNA) size ranges typically from 29 to 160 kb, and they infect bacterial phyla of Bacteroidota, Cyanobacteriota, and Pseudomonadota. Only four of the Baltic bacteriophages genomes sequenced so far belong to small tailless phages from the *Microviridae* family, characterized by ssDNA as a genetic material. They infect *Cellulophaga baltica*, and their genome size is 6.5 kb (Šulčius and Holmfeldf 2016). The largest collection of sequenced phages (121 genomes) was created by Hoetzinger et al. (2021), who isolated viruses against *Flavobacterium* sp. and *Reinheimera* sp. during a mesocosm experiment. The genomic analysis led to the identification of the newly discovered viral genus, *Immutovirus*, harboring gene sets putatively coding for proteins involved in the synthesis of modified nucleotides and glycosylation of bacterial cell surface components (Hoetzinger et al. 2021). Nilsson et al. (2019, 2020) obtained genome sequences for 54 and 38 phages infecting *Rheinheimera* sp. and *Flavobacterium* sp., respectively. Other researchers sequenced the genomes of six cold-active (psychrophilic) phages (Senčilo et al. 2015), isolated earlier from the Baltic Sea ice (Luhtanen et al. 2014). Those authors observed phage influence on the total composition of *Shewanella* host populations, and thus their impact on biogeochemical processes in this ecosystem (Senčilo et al. 2015). The genomic characterization of the cyanophage vB_AphaS-CL131, infecting a filamentous diazotrophic cyanobacterium, revealed previously undescribed features of cyanophage genomes (e.g. CRISPR–Cas and toxin–antitoxin systems), and therefore provided the new insights on the interaction of bloom-forming cyanobacteria and their viruses (Šulčius et al. 2019). Four Baltic Sea phages have recently been identified as being against bacterial microbiota members of the moon jellyfish *Aurelia aurita*. Remarkably, one of them was described as being efficient in infecting Gram-negative and Gram-positive bacterial species of genera *Pseudomonas* and *Staphylococcus*, though strains of the latter host were infected with relatively low efficiencies of plating (Stante et al. 2023). Such a broad host range is very unusual for bacteriophages, which are known to be particularly specific to their hosts, either bacterial species or even strain(s). Thus, this observation requires further confirmation, especially excluding the possibility of contamination of the tested phage lysate(s) with other phage(s).

According to the literature, the most commonly used location in the Baltic Sea for sample collection in the above studies was Linnaeus Microbial Observatory, located in the Baltic Proper (Nilsson et al. 2019, 2020, Hoetzinger et al. 2021).

However, bacteriophages were also isolated from the Gulf of Bothnia (Holmfeldt et al. 2010), Gulf of Finland (Luhtanen et al. 2014, Senčilo et al. 2015, Castillo et al. 2021), Barth Lagoon (Wolf et al. 2004), Gulf of Gdansk (Jakubowska-Deredas et al. 2012), Curonian Lagoon (Šulčius et al. 2011, 2015), and Kiel Fjord (Stante et al. 2023). Sediment samples were often collected from anoxic areas in Central Baltic, like Gotland Deep and Landsort Deep (Weinbauer et al. 2003, Cai et al. 2019, Köstner et al. 2019), the deepest point of the Baltic Sea.

Only a few authors used exclusively novel methodologies, such as metagenomics and proteomics, in phage research (Holmfeldt et al. 2013, Allen et al. 2017, Broman et al. 2021, Heyerhoff et al. 2022). There are 330 publicly available microbial metagenomes obtained from Baltic sediment and water samples in the National Center for Biotechnology Information (NCBI) sequence read archive (SRA) (https://www.ncbi.nlm.nih.gov/sra/?term=Baltic+sea+AND+viral+metagenomes; last accessed on 7 March 2024). Access to these data enables the analysis of bacteriophage composition and distribution across the Baltic Sea.

Baltic bacteriophage distribution seems to depend on such factors as water depth and access to oxygen, while their abundance is independent of the north–southerly salinity gradient spread in the Baltic Sea (Heyerhoff et al. 2022). For example, the viral and prokaryotic abundances in the Baltic Sea subseafloor sediments are similar to those in marine surface sediments. The abundances are much higher than on the deep-sea surface, which may be due to the fact that phages are better preserved and demonstrate lower decay rates at depth (Cai et al. 2019). This phenomenon was confirmed by higher virus-to-prokaryote ratios, observed in deeper sediments with fewer cells and lower organic matter content. On average, there are billions of viral particles per cm^3^ of sediment (Cai et al. 2019). Moreover, the phage composition varies depending on the depth below the sea floor. Sediment stations from the Bornholm Basin and the Bay of Aarhus sampled at depths of 0.75–3 m below the sea floor (mbsf) displayed higher counts of *Mycobacterium* phage Sparkdehlily. On the other hand, deep subsurface stations sampled at 24.1 and 67.5 mbsf, close to the island of Anholt and the Little Belt, respectively, were defined by the abundant *Ralstonia* phage RSS30 (Heyerhoff et al. 2022).

Phage abundance and composition also depend on the oxygen gradient. Generally, a higher abundance of viruses in deep anoxic water, such as dead zone sediments, in comparison to an oxygenated environment, has been reported in the Baltic Sea (Broman et al. 2021). In terms of composition, dead zone sediments contain different cyanophages than oxic sediments. Interestingly, most cyanophage contigs with a high relative abundance, generated in metagenomic analyses in the oxic sediment, belonged to the morphotype of Siphoviridae, while cyanophages in the hypoxic–anoxic sediment mainly belonged to morphotypes Podoviridae and Myoviridae (Broman et al. 2021). The difference in cyanophage alpha and beta diversity between the oxic and hypoxic–anoxic sediments may suggest that host-associated cyanobacteria capable of surviving in oxygen-deficient environments select for specific cyanophages. In terms of host taxonomic composition, based on the metagenomic analyses, *Cyanobium* was a more abundant genus in an oxic station than in hypoxic–anoxic stations, and *Synechococcus* showed the opposite pattern (Broman et al. 2021).

Metagenomic studies have extended our knowledge of the role of marine phages. Baltic Sea phages are thought to have major roles in modulating microbial communities, such as controlling prokaryotic population size, turnover rate, and diversity. Up to 20% of oceanic prokaryotic organisms are estimated to die daily due to viral infection and lysis (Broman et al. 2021). Phages are the most important cause of prokaryotic mortality in deep sediments since eukaryotic grazers (metazoan and protozoan) are thought to be absent in this habitat (Cai et al. 2019). Viral lysis of prokaryotic cells releases organic material that supports growth and nutrient recycling by noninfected cells (Cai et al. 2019). For example, viral lysis of cyanobacterial blooms increases the turnover of carbon, phosphorus, and nitrogen and their availability to other microorganisms in both oxic and anoxic zones (Allen et al. 2017, Broman et al. 2021, Heyerhoff et al. 2022). The large amounts of cyanobacteria sinking to dead zone sediments are known to fuel the benthic ecosystems with phosphorus (so-called internal loading) (Brorman et al. 2021). Genomes of Baltic bacteriophages frequently contain auxiliary metabolic genes (AMGs), which allow phages to augment the metabolism of their hosts or enhance virus fitness (Broman et al. 2022). Acquiring AMGs during viral infections may improve bacterial adaptation to various ecosystem fluctuations, such as temperature, organic matter concentration, salinity, or redox regimes (Heyerhoff et al. 2022). Examples of AMGs found in Baltic Sea cyanophages are photosynthesis-related genes, *psbA* and *cobS*, or genes for the phosphorus regulon *phoH*. The function of *phoH* is still poorly understood. However, such phages may influence the cycling of phosphate by infecting cyanobacteria in the sediment (Broman et al. 2017). Another example of AMGs found in Baltic phages is the *cobS* gene, encoding a protein catalysing the final step in the bacterial cobalamin (vitamin B12) biosynthesis (Magnúsdóttir et al. 2015). However, any speculations about the involvement of viruses in cobalamin biosynthesis in the pelagic ecosystem require more targeted analyses and experimental evidence (Heyerhoff et al. 2022). Furthermore, viruses from the water columns procure AMGs specific for photosynthesis, while viruses in sediments acquire AMGs that are part of the nutrient cycling pathways, such as sulfur cycling. This also correlates with the salinity gradient stretching by depth due to the higher density of saline water (Heyerhoff et al. 2022). On the other hand, viruses use AMGs to evade host restriction mechanisms by modifying their DNA through methylation or utilization of PreQ_0_ (7-cyano-7-deazaguanine, a specific base being the biosynthetic precursor of queuosine-tRNA). These DNA modification AMGs were found to be highly abundant in the Baltic Sea and have also been observed to be globally conserved (Heyerhoff et al. 2022).

Baltic Sea phages may also increase host genetic diversity. The presence of a phage integrase gene in a cryptic plasmid pSFKW33 from the psychrotrophic bacterium *Shewanella* sp., an isolate from the Gulf of Gdansk (the Baltic Sea), was detected by Werbowy et al. (2009). This suggests the possibility of the horizontal transfer of the phage integrase gene to the cryptic DNA plasmid pMP1 from the marine environment (Werbowy et al. 2009). Apart from that, the effect of cyanophage infection and lysis on the dynamics of the hepatotoxin nodularin (NOD) has been studied to demonstrate the importance of such infection. It influenced the population toxicity of filamentous cyanobacteria and demonstrated a significant contribution of virus-mediated cell lysis to converting NOD from the particulate to the dissolved phase (Šulčius et al. 2018). Another example of how Baltic Sea phages can impact the ecology of the marine system was a demonstration that the gene encoding the outer membrane iron receptor protein was detected in phage metagenomic reads as the gene with the highest temporal allele variability (Beier et al. 2020). Moreover, the corresponding protein represented a putative target for phage infection, consistent with the “Trojan Horse Hypothesis.” If this hypothesis is true, phages incorporate iron atoms into their tail to be recognized and enter host cells via K02014 transporters (Bonnain et al. 2016).

Finally, bacteriophages are known to modulate community structure through infection cycles (Allen et al. 2017). The metagenomic analyses revealed that the lytic life mode is predominant in the Baltic Sea, possibly due to high nutrient availabilities (Heyerhoff et al. 2022). On the other hand, in the sediments, the average inducible lysogenic viral production accounts for about 20% of the total potential viral production, indicating that lysogenic infection might be an important, but not a predominant, life cycle of viruses in the sediments (Cai et al. 2019).

### Viruses infecting eukaryotic organisms occurring in the Baltic Sea

The number of reports on viruses infecting eukaryotic organisms in the Baltic Sea is significantly lower than that describing bacteriophages. Nevertheless, the presence of viruses in the Baltic Sea was noted almost half a century ago (Steinman 1977), though that description was very general, similar to the paper published 1 year later (Gärtner 1978).

The primary characterization of viruses infecting eukaryotes from the Baltic Sea was possible only after the introduction of modern genomic methods. In fact, until 2016, there were only single reports on such viruses, as summarized by Šulčius and Holmfeldt (2016). To that time, among these viruses, only those infecting algal and protist cells belonging to *Mantoniella* sp. and *Micromonas pusilla* were identified; most of them were from the family *Phycodnaviridae*. These algal and protist viruses develop quite slowly compared to bacteriophages, with infection cycles lasting a week or so (Sahlsten 1998, Eissler et al. 2003). Eukaryotic cell-specific viruses from the Baltic Sea were ascribed as representing potentially high variability, though some are specific to individual strains (Brussaard et al. 2004, Martinez et al. 2015).

Reports on the isolation and identification of specific viruses infecting marine animals in the Baltic Sea are especially scarce. However, some interesting reports were published, indicating specific viral infections of harbor seals by Phocine Distemper Virus (PDV; this virus caused several thousand deaths of seals in 1988 and 2002), or of sea eagles by Influenza A virus (IAV), as summarized by Sonne et al. (2020). Indeed, screening for PDV and IAV infecting seals and porpoises in the Baltic Sea indicated that these viruses are relatively rare in this environment (Stokholm et al. 2023). Nevertheless, viral infections of animals living in the Baltic Sea can be severe, as indicated by the abovementioned disease of harbor seals caused by PDV or a more recently reported occurrence of a highly pathogenic strain of IAV, identified as H5N8 in gray seals (Shin et al. 2019).

Only recent studies, in which advanced transcriptomic and metagenomic methods were employed, allowed to confirm that the variability of eukaryote-specific viruses is high, while our knowledge of their specificity and characteristic features is very incomplete. Metagenomic and metatranscriptomic analyses performed with samples from several stations located in the Baltic Sea indicated that viruses infecting phytoplankton might be the largest group among the eukaryotic host-specific viruses (Zeigler et al. 2017). However, the presence of viruses from various families was demonstrated, including retroviruses, picornaviruses, parvoviruses, and others. According to analyses of nucleic acid sequences, it could be concluded that viruses present in the Baltic Sea should be able to infect fish, tetrapods, and insects. In addition, sequences derived from porcine parvoviruses, along with human viruses, like picobirnavirus, norovirus, hepatitis E virus, coronavirus, and rhinovirus A, were detected, demonstrating that the Baltic Sea virome is contaminated by viruses originating from urban and/or industrial regions (Zeigler et al. 2017).

Recent metagenomic analyses confirmed that bacteriophages, not viruses infecting eukaryotic cells, are the most abundant group of viruses occurring in the Baltic Sea (Heyerhoff et al. 2022). Interestingly, viruses infecting dinoflagellates and diatoms reported previously to occur frequently in some other seas (Nagasaki 2008, Tomaru et al. 2009), were under-represented in samples from the Baltic Sea. Nevertheless, the presence of other groups of viruses in this basin has been confirmed, including phycodnaviruses, baculoviruses, poxviruses, herpesviruses, and mimiviruses (Heyerhoff et al. 2022).

**Figure 8.**
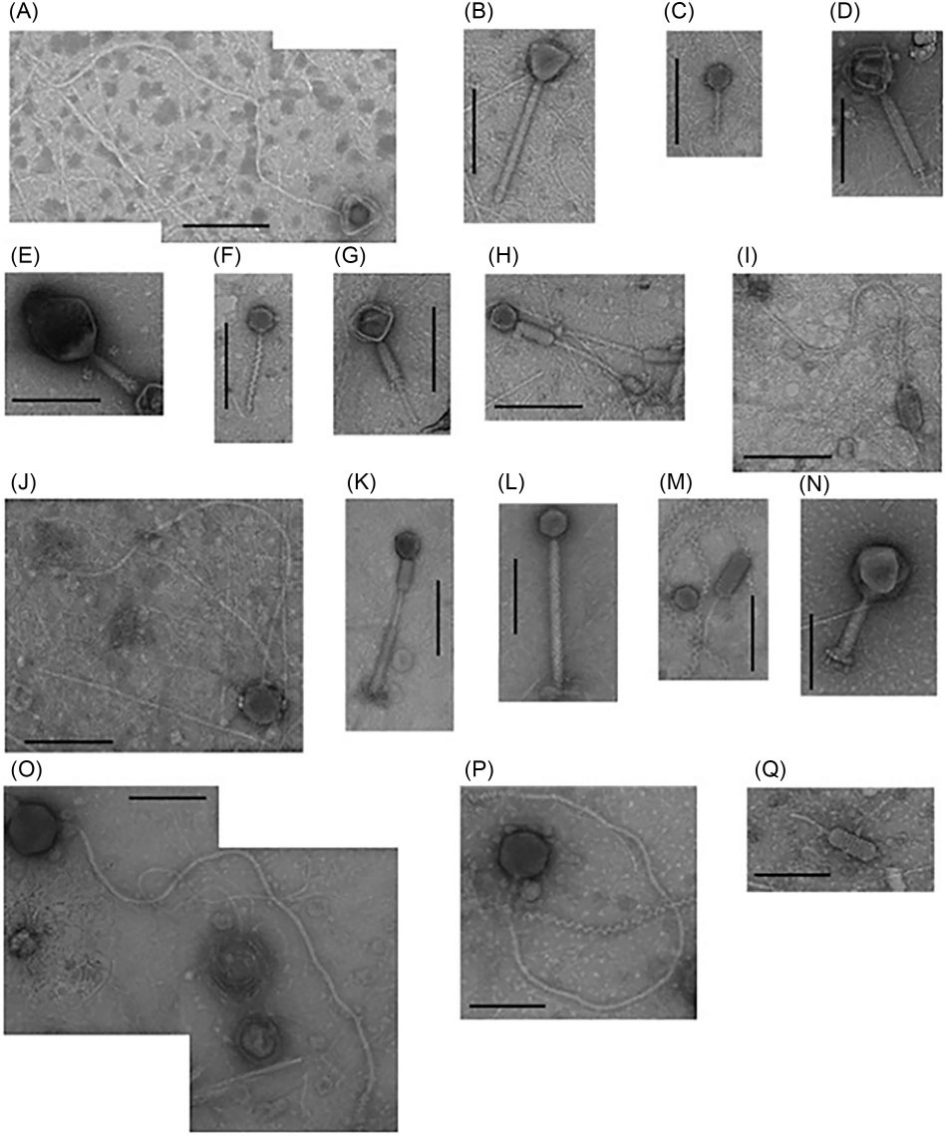
Electron microphotographs of bacteriophages from Baltic Sea samples. Several morphotypes are shown, including myoviruses (B, D, E, G, H, K, L, and N), podoviruses (M), and siphoviruses with either short (C, F, M, and Q) or extremely long tails (A, J, O, and P), and prolate heads (I, M, and Q). The bar corresponds to 200 nm. Reproduced from Jakubowska-Deredas et al. (2012), with permission of the publisher (Elsevier Masson SAS).

In summary, bacteriophages are a predominant group of viruses occurring in the Baltic Sea. Examples of electron micrographs of bacteriophage virions from the Baltic Sea are shown in Fig. 8. Among viruses infecting eukaryotic hosts, *Phycodnaviridae* appears to be the most abundant group. Nevertheless, many other groups of viruses could be detected in this sea, including those pathogenic to fish and insects, but also pigs or humans. The presence of those viruses indicates that the Baltic Sea virome is contaminated with viruses originating from urban and/or industrial habitats.

## Biological contaminations in the Baltic Sea

The Baltic Sea is especially sensitive to different contaminations, as a semienclosed sea with estuaries of large rivers (like the Vistula and the Oder), a highly populated and industrialized catchment area, and limited water influx from the oceans (Couper and Mutton 2019, Gyraite et al. 2019). These include chemical, physical, and biological contaminants from natural sources and urban/industrial environments. While chemical and physical contaminants change the environmental conditions and affect organisms directly or indirectly, the presence of biological entities that appear randomly in the Baltic Sea (so-called biological pollution) significantly influences the composition of the microbial community of this habitat. In this chapter, we will discuss sources of biological pollution and biological contamination, paying particular attention to antibiotic-resistant bacteria, which may have biological significance and cause a risk of spreading this feature, which is dangerous to human health.

### Sources of potential biological pollution

The potential sources of the Baltic Sea contamination are microbiological factors such as bacteria, viruses, parasites, algae, and the so-called sanitary contamination. The role in this process is played by sewage discharged directly into the sea, bypassing river runoff, and mainly by rivers that belong to dynamic ecosystems (Jutterström et al. 2014). More than 250 streams and rivers flow into the Baltic Sea, giving an annual flow of about 15 000 m^3^/s (Jutterström et al. 2014). Municipal sewage and stormwater are serious water pollutants (Berndtsson and Paul 2015). Rainwater collects pollutants from agricultural land fertilized with manure, liquid manure, and synthetic fertilizers. Net precipitation over the Baltic Sea adds about 1000 m^3^/s of annual flow.

There are emergency discharges of raw sewage from treatment plants (Michalska et al. 2019). The Baltic Sea has also been polluted by fuel leaked from wrecks after World War II and today by millions of liters of sewage from passenger ships (pollution generated at sea). In addition to water pollution, depending on the meteorological conditions, bioaerosols are formed, which can contain a wide variety of bacteria, e.g. *Staphylococcus* sp., *Bacillus* spp., *Pseudomonas aeruginosa, E. coli*, or other Enterobacteriaceae, among others (Michalska et al. 2019). Significantly higher concentrations of psychrophilic, mesophilic, and coliform bacteria were observed in seawater and air from the Gulf of Gdansk after emergency discharges of raw sewage in 2018 (Michalska et al. 2019). Therefore, not only the water body but also the areas surrounding it can pose a threat to human and animal health (Sonne et al. 2020).

### Biological pollutants (contaminations)

In the sea, there are autochthonous and allochthonous microbes. *Escherichia coli* is a fecal bacterium recognized as an indicator of environmental contamination (Schippmann et al. 2013). Its occurrence is most often temporary; however, beach recreational facilities are usually closed due to their presence. The Oder (Szczecin) Lagoon on the southern Baltic coast was examined for *E. coli* contamination by Gotkowska-Płachta et al. (2016). The authors suggested that insufficient wastewater treatment in Szczecin is a major source of fecal contamination, even on beaches located up to 20 km downstream. Moreover, southwest winds transport *E. coli* along the east coast and favor high titers of this bacterium on beaches. The concentrations of Enterobacteriaceae and *E. coli* (fecal indicator bacteria) depend on water temperature, chemical oxygen demand, dissolved oxygen (DO), ammonia nitrogen (NH_4_-N), nitrite nitrogen (NO_2_-N), and total phosphorus (Gotkowska-Płachta et al. 2016).

Sea water and beach sand, especially in water bathing areas, pose a potential threat to human health (Mancini et al. 2005, Bonilla et al. 2007). In many reports, pathogenic bacteria (for example, *S. aureus, Klebsiella pneumoniae, P. aeruginosa, Shigella* spp., *Salmonella enterica, Campylobacter* spp., *Neisseria* spp., *Clostridium perfringens*, and *Aeromonas hydrophila*) have been described in the water and beach sand (Kueh et al. 1995, Gabutti et al. 2000, Elmanama et al. 2005, Mancini et al. 2005, Bonilla et al. 2007, Heaney et al. 2009).

Warm waters (>18°C) and low salinity (<2.5% NaCl) waters offer optimal functioning conditions for the growth of *Vibrio* spp. (Gyraite et al. 2019). The mean temperature of the Baltic Sea ranges from −10°C to 17°C, depending on the region. Still it is estimated that global warming may have a significant impact on the prevalence of *Vibrio* spp. in the Baltic Sea region (Jutterström et al. 2014). The results of that study indicated that the total abundance of *Vibrio* spp. detected in the coastal waters of Lithuania (10^2^–10^4^ CFU/l) is very similar to the abundance of *Vibrio* spp. in the coastal waters of Poland (2.11 × 10^4^ CFU/l) (Mudryk et al. 2014) and the brackish waters (0.7–1.5 PSU) of the Netherlands (Ijsselmeer—levels of 10^4^ CFU/l).

It was suggested that green algae and cyanobacteria, as well as lower salinity, play a role in the growth and spread of *Vibrio* spp. The increase in dissolved organic matter resulting from intense phytoplankton blooms can remarkably support the growth of *Vibrio*, and cyanobacterial-derived organic matter can be more important than temperature to determine the total abundance of *Vibrio* within a temperature range of 12°C–25°C (Eiler et al. 2007). A recent study, which covered the salinity and eutrophication gradients of the Baltic Sea, revealed that the most reliable predictors of pathogenic bacterium *Vibrio vulnificus* presence were features related to eutrophication, such as particulate organic carbon and nitrogen, as well as the occurrence of potential phytoplankton blooms and associated species. Therefore, reducing nutrient inputs could effectively control *V. vulnificus* populations in eutrophied brackish coasts (Riedinger et al. 2024).

In some systems, the presence of *Vibrio* spp. can be associated with dinoflagellate blooms (Greenfield et al. 2017). The bloom can also affect the survival rate of cultivable forms of these bacteria (Islam and Tanaka 2004). Moreover, a study on the fate of allochthonous bacteria (of fecal origin) indicated that the high turbidity and the amount of suspended organic matter in the Curonian Lagoon could support bacterial survival and proliferation (Kataržytė et al. 2018). The combination of the naturally low salinity of Baltic waters and the rising annual sea surface temperature also play significant roles (Schets et al. 2011).

Pathogenic bacteria present in the sea can cause gastroenteritis, hepatitis, meningitis, respiratory diseases, skin, eye, ear, or nose infections and the appearance of dermatitis and mycosis. Mudryk et al. (2014) investigated the presence of *Aeromonas, P. aeruginosa, Staphylococcus*, and *Vibrio*-like organisms on beach sand. Bacteria belonging to *Aeromonas* were more prevalent, while bacteria of the genus *Staphylococcus* were less abundant (Mudryk et al. 2014). Dry sand was colonized by the highest number of potentially pathogenic bacteria. Differences in their abundance were observed between the surface and near-surface sand layers, with a marked decrease toward the deeper layers.

In addition, wild animals, especially aquatic and terrestrial ones, are important reservoirs of many pathogenic microorganisms. Waterfowls are vectors transmitting many pathogenic microorganisms from the human environment (hospital environments, sewage treatment plants, and landfill sites) (Rybak et al. 2022). Marine mammals are frequently considered good sentinels for human, animal, and environmental health due to their long lifespan, coastal habitat, and characteristics as predators of the upper chain. Marine mammals can provide information that helps to improve the understanding of the health of the marine and coastal environment. Unfortunately, marine animals become victims of biological and chemical contaminants. Sonne et al. (2020) have indicated pathogens responsible for bacterial (*Brucella* spp., *C. perfringens, E. coli, E. rhusiopathiae*, β-haemolytic streptococci, and *S. aureus*), viral (influenza A), and parasitic diseases (*Giardia, Cryptosporidium*, and *Toxoplasma*) (Gabutti et al. 2000, Cabezón et al. 2011, Sonne et al. 2020, Fayer 2004, Reboredo-Fernández et al. 2015). They were isolated from animal species recognized as indicators of ecosystem condition: ringed seal (*Pusa hispida*), common seal (*Phoca vitulina* and *Phoca hispida*), grey seal (*Halichoerus grypus*), harbor porpoise (*Phocoena phocoena*), white-tailed eagle (*Haliaeetus albicilla*), eider (*Somateria mollissima*), and pink-fronted goose (*Anser brachyrhynchus*) (Siebert et al. 2009, Sonne et al. 2020). Autopsies performed on porpoises found on the coasts of Latvia, Poland, Germany, and Denmark showed a significantly higher prevalence of lung inflammatory lesions and parasitic infections compared to control populations from Arctic waters (Ma et al. 2020, Schmidt et al. 2020, Siebert et al. 2020, Dietz et al. 2021). Parasites, such as *C. semerme* and *P. truncatum*, present in the colon and liver of Baltic gray seals, respectively, and anisakis nematodes, require particular monitoring due to their effects on animal health. Additionally, the distribution of existing viral and bacterial pathogens, and the emergence and spread of new pathogens, must be monitored to assess the health status of key Baltic species (Sonne et al. 2020, Desforges et al. 2022).

### Problems of antibiotic-resistant bacteria

In marine waters and sediments, two sources of antibiotic-resistant bacteria and resistance genes can be identified: one from terrestrial bacteria originating from anthropogenic activities of the surrounding environment and the other from indigenous estuarine or coastal marine bacteria. Both groups of bacteria can enter seawater with antibiotic-resistant plasmids, which can be responsible for the observed prevalence of resistance genes in the marine environment (Tendencia and de la Pena 2001, Dang et al. 2008, de Oliveira and Pinhata 2008, Mudryk et al. 2010).

In the past few decades, the uncontrolled use of pharmaceutical substances (mainly antibiotics, but also including anti-inflammatory, analgesic, cardiovascular, and central nervous system agents) in human and veterinary medicine, animal husbandry, agriculture, and aquaculture has caused an increased introduction of those agents into the aquatic environment (Hirsch et al. 1999, Metcalfe et al. 2003, Dang et al. 2006). Many reports have shown the presence of bacteria resistant to numerous antibiotics in both deep-sea water and bottom sediments on the seabed and beach sand, as a result of pharmaceutical contamination of natural surface water or wastewater treatment plant effluent. Environmental bacteria may play a role as reservoirs of antibiotic resistance, and resistance genes are also exchanged by different freshwater and marine bacteria.

Treated and untreated sewage, hospital waste, and agricultural runoff are also responsible for spreading multidrug resistance in marine and freshwater ecosystems. There are indications that wastewater treatment, although generally reducing the number of bacteria, might lead to an accumulation of resistance genes. As noted by Hsu et al. (1992), differences in the rates of resistance of bacteria to various antimicrobials may reflect the history of antimicrobial use, so it may be possible to use resistance of bacteria as an indicator of antimicrobial use (Mudryk et al. 2010). It was demonstrated that bacteria isolated from the Czołpino sand beach (Polish coast of the Baltic Sea, the Slovincian National Park) can be characterized by low levels of antibiotic resistance and large differences in the resistance levels to particular antibiotics tested. Among all isolated strains, the high resistance frequency (20%–27%) was related to amoxicillin, amoxicillin/clavulanic acid, cefaclor, cefuroxime, clindamycin, ciprofloxacin, erythromycin, and penicillin. Bacterial resistance to these antibiotics was also noted in other marine beaches (Mudryk 2005, de Oliveira and Pinhata 2008), in coastal waters and marine sediments (Hermansson et al. 1987, Dumontent et al. 2000, Harakeh et al. 2006), and in marine aquaculture (Akinbowale et al. 2006, Mudryk et al. 2010). Moskot et al. (2012) have also demonstrated the association of resistance genes for different classes of antibiotics among bacteria isolated from the surface waters of the Baltic Sea. The isolated bacteria belonged mainly to Gammaproteobacteria and accounted for up to 78% of the studied isolates. Relationships were found between resistance to ampicillin and erythromycin, chloramphenicol and erythromycin, chloramphenicol and tetracycline, and erythromycin and tetracycline, but these strains contained numerous resistance genes not associated with the presence of plasmids.

Wildlife is a reservoir and vector for antibiotic-resistant bacteria and resistance genes (Rybak et al. 2022). Birds play an important role, as their feces were found to be another important source of such bacterial contamination in natural water bodies. Birds themselves are likely to acquire resistant bacteria from the environment, indicating the complex transmission dynamics of antimicrobial resistance (Hooban et al. 2020, Edge and Hill 2005).

Rybak et al. (2022) analysed cloacal swabs of wild birds that lived, among others, in northern Poland, including municipal beaches on the Baltic Sea coast. They detected avian *E. coli* ESBL/AmpC strains with *bla*_CTX-M_, *bla*_TEM_, *bla*_SHV_, and *bla*_AmpC_ genes in various phylogenetic groups (A, B1, B2, and D) of *E. coli*. The phylogenetic pathogenic B2 group was not the most prevalent one, but in the case of this group, the coexistence of most ESBL genes was observed. Phylogenetic group A (39.4%), considered a commensal pathotype, was the most represented in the environment, and in addition to antibiotic resistance, showed high virulence [iron acquisition systems (93.9%) and autotransporters (60.6%), typical of pathogens]. It indicates that wild birds on the beach can be a reservoir of ESBL/AmpC *E. coli* carrying different virulence factors.

Gross et al. (2022a) showed that also marine mammals of the North and Baltic Seas serve as reservoirs and vectors for antimicrobial-resistant bacteria, thus participating in the circulation of resistant bacteria and resistance genes. The harbor porpoises (*P. phocoena*) mainly carried *E. coli* isolates belonging to phylogenetic group B1, while seal isolates were most frequently assigned to group B2. The presence of resistant *E. coli* in 39.4% of the marine mammal samples was confirmed, while no resistant isolates were obtained from any of the fish samples. Gross et al. (2022b) also described the dispersal of antimicrobial-resistant bacteria in wild birds in Germany, thereby allowing conclusions on the degree of environmental contamination and potential public health concerns. More than half of the isolates belonged to the phylogenetic group B1. Of all isolates, 24.4% were classified as APEC isolates, of which almost 82% were identified as multidrug-resistant (Gross et al. 2022b). As a consequence of the widespread use of antibiotics by humans, a dramatic and global increase in the number of antibiotic-resistant bacteria, multiple antibiotic resistance, pathogenic bacteria resistance, and reduced efficacy of antibiotic treatment for diseases caused by resistant pathogens are observed in aquatic environments. At the same time, an increase in antibiotic concentrations in aquatic ecosystems can generate new selective pressures on natural bacterial populations (Mudryk et al. 2010).

The occurrence of multidrug-resistant isolates is of particular concern for human and domestic animal health. To combat antimicrobial resistance, surveillance of different ecosystems is essential by monitoring important reservoir and vector species. The Baltic Sea is a unique example of this problem, as it is especially prone to biological contamination and the spreading of specific features, like antibiotic resistance.

## Concluding remarks

The Baltic Sea is a particular habitat for microorganisms. It is a semienclosed, shelf sea connected to the ocean only through narrow and shallow waters. Therefore, its topography and hydrographic settings are quite unusual for marine environments. Many factors, like eutrophication, deoxygenation, acidification, and anthropogenic pollution influence the biogeochemical properties of the waters. All these conditions influence Baltic microorganisms significantly, affecting their composition, diversity, and abundance. Among the most important factors modulating the Baltic microbiome are surface salinity gradient (from marine to nearly brackish or freshwater), reduced circulation and stratification, which cause hypoxic and anoxic conditions, high sedimentation rates, and inflow of terrestrial organisms. The Baltic Sea is also especially sensitive to chemical, physical, and biological contaminations that accumulate effectively in this semienclosed sea, and strongly influence all components of the microbiota. The characteristic features of different groups of microbiota occurring in the Baltic Sea, together with environmental conditions shaping microbial diversity and identified research gaps, are summarized in Table 1.

**Table 1.** Characteristic features of different groups of microorganisms occurring in the Baltic Sea.

Microorganism group	Dominant taxonomical groups	Environmental conditions shaping microbial diversity	Used novel methods in biodiversity research and target regions	Functional roles	Investigated associations with other organisms	Identified research gaps
Archaea	Bathyarchaeota; Euryarchaeota marine group II; Methanomicrobia; Thaumarchaeota	Salinity; nutrient availability; pH; temperature; oxygen levels; redox stratification; depth	Genomic sequencing; metagenomic and metatranscriptomic analyses; qPCR; metabolomics	Chemotrophs; nonoxygenic phototrophs; saprophytes	None	Many DNA sequences present in databases, which are not assigned to any known groups of archea
Bacteria (excluding cyanobacteria)	Pelagibacterales (an Alphaproteobacterial clade SAR11-IIIa; pelagic); Epsilonproteobacteria (benthic); Chloroflexota; Desulfobacterota; Caldatribacteriota; Acidobacteriota; Planctomycetota; Pseudomonadota; *S. baltica; Rheinheimera* sp. BAL341; *E. coli* and other fecal coliforms (due to urban/industry-derived contamination)	Salinity (the crucial condition); nutrient availability; pH; temperature; oxygen levels; redox stratification; depth; occurrence of cyanobacteria	Ultra-deep sequencing; CARD-FISH; pyrosequencing; T-RFLP; metagenomic and metatranscriptomic analyses; qPCR; metabolomics	Saprophytes; symbionts; parasites/pathogens	Fish (cod, *G. morhua*; European plaice, *P. platessa*); copepods (*Temora* sp., *Acartia* sp., and others); sponge (*H. panicea*); the brown algae (*L. saccharina*); bryozoans; *F. vesiculosus; D. sanguinea*; soft coral (*A. digitatum*); *N. spumigena*; mussels (*Mytilus* spp.); ascidian (*C. intestinalis*); European plaice (*P. platessa*); herring (*C. harengus*); *Ulva* sp.; humans	Many DNA sequences present in databases, which are not assigned to any known groups of bacteria
Cyanobacteria	PCy (mainly *Synechococcus* spp. and *Cyanobium* spp., as for gene abundance)	Temperature (the predominant condition); salinity; depth	Barcoding and metabarcoding; 16S rRNA sequencing; metagenome sequencing; qPCR; mass spectrometry; HTS	OPs	Bacteria and archea	The lack of reliable databases containing well-annotated genomes of cyanobacteria
Protists	Diatoms (Bacillariophyceae; a highly dominant group) (*Gedaniella* spp., *P. baltica, Planothidium* sp., *S. marinoi*); dinoflagellates (Dinophyceae); ciliates (Ciliophora)	Light availability; salinity; temperature; nutrients	18S rRNA sequencing; DNA barcoding and metabarcoding; imaging in-flow systems, including IFCB	OPs (diatoms, some dinoflagellates); saprophytes; parasites (various protists)	None for phototrophs; shellfish and fish for parasites	Scarcity of molecular data (including DNA sequences)
Fungi	Rozellomycota (pelagic); Chytridiomycota (benthic, pelagic realm); Ascomycota	Salinity (pelagic at <8 PSU more freshwater, >8 marine community; benthic); nutrients (coastal, benthic); depth (benthic)	HTS; metabarcoding (long-read 18S-ITS1-5.8S-ITS2-28S rRNA); fungal cell wall markers; CFW and WGA (for Chytridiomycota)	Parasites/pathogens; saprophytes; symbionts	Algae (*Fucus*), seagrass (*Z. marina*) tunicates (*C. intestinalis*) fish (*P. platessa*)	The lack of annotated reference sequences; fungal microparasites’ role in microorganisms-related carbon flow; contribution to benthic-pelagic recycling of nutrients; lack of detailed taxonomic information for most Cryptomycota
Viruses	Bacteriophages (mainly Caudoviricetes); *Phycodnaviridae* (among viruses infecting eukaryotic organisms)	Water depth; oxygen levels; nutrient availability for host organisms; salinity; pH; temperature; redox stratification	Metagenomics; proteomics; FC; electron microscopy	Obligatory parasites	Prokaryotic organisms for bacteriophages; many different kinds of eukaryotic organisms for other viruses	Scarcity of viral genomic sequences in databases (relative to the abundance of these microbes); contamination by viruses originating from urban and/or industrial regions

**Figure 9.**
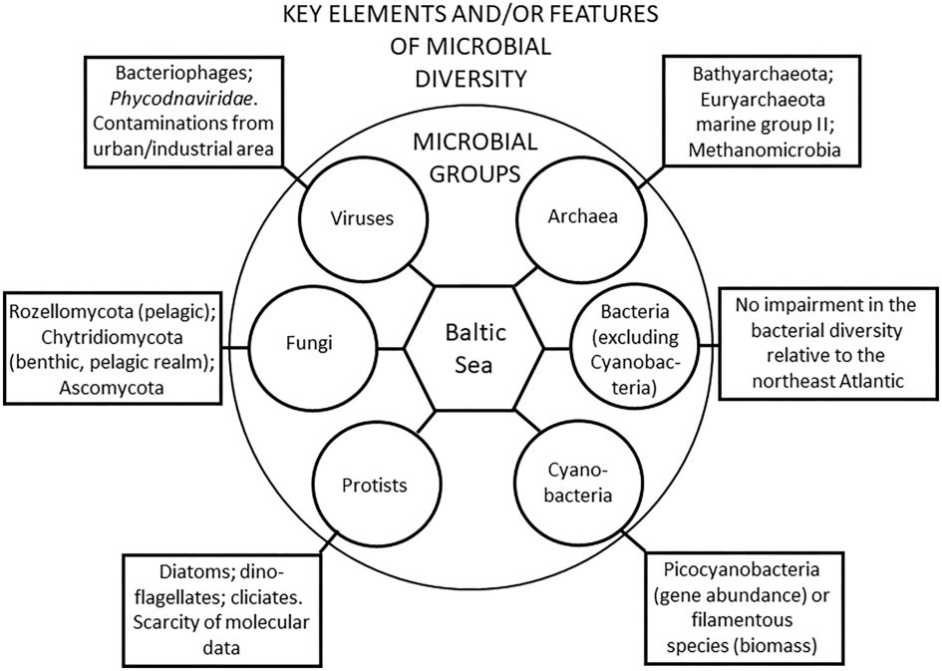
The key elements and features of the unique diversity of Baltic microorganisms. A Cyske rosette chart showing major groups of Baltic microorganisms, indicating key elements and/or features of microbial diversity among each group.

As presented and discussed in this review article, specific environmental conditions in the Baltic Sea result in highly pronounced specificity of microbial communities. Unique characteristics of the diversity of microbes in the Baltic Sea encompass the following points. First, although the low salinity causes a reduction in the biodiversity of multicellular species in the Baltic Sea relative to the populations of the North–East Atlantic, no such impairment in the bacterial diversity can be observed. Second, among cyanobacteria, the picocyanobacterial group dominates when considering gene abundance, while filamentous cyanobacteria dominate by means of biomass in the Baltic Sea. Third, recent analyses indicated that the diversity of diatoms and dinoflagellates in the Baltic Sea is significantly larger than described a few decades ago, but the problem is that molecular studies on these groups of organisms are scarce. Fourth, even deeper knowledge gaps on communities of other protistan groups in the Baltic Sea are evident. Fifth, fungi are another group of under-investigated microorganisms in the Baltic Sea; however, one can conclude that despite the main factors controlling fungal diversity in coastal ecosystems, including salinity, DO, and nutrient conditions, the salinity is a crucial pelagic fungal community composition parameter in the assessed marine environment. Sixth, bacteriophages are a predominant group of viruses in the Baltic Sea, while among viruses infecting eukaryotic hosts, *Phycodnaviridae* are the most abundant. It is also worth noting that the Baltic Sea virome is contaminated with viruses from urban and/or industrial habitats. The key points of the unique diversity of Baltic microorganisms are summarized in the form of the Cyske rosette chart (according to Cyske et al. 2022), presented in Fig. 9.

Finally, when investigating microbiomes of specific habitats, like the Baltic Sea, it is crucial to indicate that molecular methods provide a powerful tool for studies on the biodiversity of microorganisms. However, such methods should be combined with biological investigations to obtain a comprehensive picture of the microbial diversity of the Baltic Sea, which is sufficiently different from other marine environments to be especially prone to potential errors resulting from a scarcity of data (present in various databases) related to unique properties, like very specific genes and other parts of genomes.

## Supplementary Material

fuae024_Supplemental_File
