# Correction to: Biodiversity of microorganisms in the Baltic Sea: the power of novel methods in the identification of marine microbes

**DOI:** 10.1093/femsre/fuaf002

**Published:** 2025-01-23

## Abstract

This review discusses the contribution of phenotypic heterogeneity in fungi to pathogenesis and antifungal drug resistance.

This is a correction to: Hanna Mazur-Marzec *et al*., Biodiversity of microorganisms in the Baltic Sea: the power of novel methods in the identification of marine microbes, *FEMS Microbiology Reviews*, Volume 48, Issue 5, September 2024, fuae024, https://doi.org/10.1093/femsre/fuae024.

Details of funding have been updated to include grant 2019/34/E/ST10/00217, omitted from the originally published version.

