# Supplementary material for: Biodiversity of microorganisms in the Baltic Sea: the power of novel methods in the identification of marine microbes
Source: FEMS Microbiol Rev. 2024 Oct 4;48(5):fuae024. doi: 10.1093/femsre/fuae024 (PMC11500664; doi:10.1093/femsre/fuae024)
Supplement: fuae024_Supplemental_File [file fuae024_supplemental_file.pdf]

From: PermissionsFrance <permissionsfrance@elsevier.com>
Sent: Tuesday, 20 August 2024 16:51
To: Agata Jurczak-Kurek <agata.jurczak-kurek@ug.edu.pl>
Subject: RE: Picture reprint from the article

Dear,

As per your request below, we hereby grant you permission to reproduce the material detailed in your request in print and electronic formats at no charge subject to the following conditions:

1. If any part of the material to be used (for example, figures) has appeared in our publication with credit or acknowledgement to another source, permission must also be sought from that source. If such permission is not obtained then that materials may not be included in your publication.

2. Any modification of the material is likely to harm the moral right of the authors and therefore should be first submitted and approved by the authors who are the sole owner of the moral right.

3. Suitable and visible acknowledgement to the source must be made, either as a footnote or in a reference list at the end of your publication, as follows:
   "Reproduced from *Authors name*. *Article title*. *Journal title year; volume number(issue number):first page-last page*. Copyright © *year [if applicable: name of learned society,* published by*]* Elsevier Masson SAS. All rights reserved."
4. This permission is granted for non-exclusive world rights, for the sole purpose described in your request.

Yours sincerely,
Permissions France

From: Agata Jurczak-Kurek <agata.jurczak-kurek@ug.edu.pl>
Sent: Tuesday, August 6, 2024 5:13 PM
To: PermissionsFrance <permissionsfrance@elsevier.com>
Subject: Picture reprint from the article

Dear Sir or Madam,

As a co-author of the article: "Diversity of tailed phages in Baltic Sea sediment: large number of siphoviruses with extremely long tails" published in Research in Microbiology in 2012 (Volume 163, Issue 4, May 2012, Pages 292-296, pdf version attached) I would like to ask about permission to reprint the images from the article, specifically a few microphotographs of bacterial viruses from the Figure 1.

I need these images for the next article I co-authored: "Biodiversity of microorganisms in the Baltic Sea", submitted in FEMS Microbiology Reviews, that is currently undergoing minor revision (Manuscript id: FEMSRE-24-04-0008).

I would be very grateful if you kindly let me know if such reprint is possible. Due to our upcoming submission deadline (the end of August) I need this information as soon as possible.

Yours faithfully,

Agata Jurczak-Kurek

Pozdrawiam | Best regards

dr Agata Jurczak-Kurek

Adiunkt | Assistant professor

Katedra Genetyki Ewolucyjnej i Biosystematyki |

Department of Evolutionary Genetics and Biosystematics

Wydział Biologii | Faculty of Biology

UNIWERSYTET GDAŃSKI | UNIVERSITY OF GDAŃSK

ul. Wita Stwosza 59/B306

80-308 Gdańsk | Poland

T: +48 58 523 60 95

E: agata.jurczak-kurek@ug.edu.pl

W: genetyka.ug.edu.pl

Uniwersytet Gdański | Uczelnie Fahrenheita
